# REBUS and the Anarchic Brain: Toward a Unified Model of the Brain Action of Psychedelics[Fn FN4]

**DOI:** 10.1124/pr.118.017160

**Published:** 2019-07

**Authors:** R. L. Carhart-Harris, K. J. Friston

**Affiliations:** Centre for Psychedelic Research, Division of Brain Sciences, Imperial College London, London, United Kingdom (R.L.C.-H.); and Institute of Neurology, Wellcome Trust Centre for Neuroimaging, University College London, London, United Kingdom (K.J.F.)

## Abstract

This paper formulates the action of psychedelics by integrating the free-energy principle and entropic brain hypothesis. We call this formulation relaxed beliefs under psychedelics (REBUS) and the anarchic brain, founded on the principle that—via their entropic effect on spontaneous cortical activity—psychedelics work to relax the precision of high-level priors or beliefs, thereby liberating bottom-up information flow, particularly via intrinsic sources such as the limbic system. We assemble evidence for this model and show how it can explain a broad range of phenomena associated with the psychedelic experience. With regard to their potential therapeutic use, we propose that psychedelics work to relax the precision weighting of pathologically overweighted priors underpinning various expressions of mental illness. We propose that this process entails an increased sensitization of high-level priors to bottom-up signaling (stemming from intrinsic sources), and that this heightened sensitivity enables the potential revision and deweighting of overweighted priors. We end by discussing further implications of the model, such as that psychedelics can bring about the revision of other heavily weighted high-level priors, not directly related to mental health, such as those underlying partisan and/or overly-confident political, religious, and/or philosophical perspectives.

**Significance Statement:**

Psychedelics are capturing interest, with efforts underway to bring psilocybin therapy to marketing authorisation and legal access within a decade, spearheaded by the findings of a series of phase 2 trials. In this climate, a compelling unified model of *how* psychedelics alter brain function to alter consciousness would have appeal. Towards this end, we have sought to integrate a leading model of global brain function, *hierarchical predictive coding*, with an often-cited model of the acute action of psychedelics, *the entropic brain hypothesis*. The resulting synthesis states that psychedelics work to relax high-level priors, sensitising them to liberated bottom-up information flow, which, with the right intention, care provision and context, can help guide and cultivate the revision of entrenched pathological priors.

## I. Introduction

Psychedelic (mind-manifesting) drugs such as d-lysergic acid diethylamide (LSD) and psilocybin are capturing people’s imagination and permeating popular culture on a scale not seen since the 1960s (Hanks, 2010; Keshavan and Sudarshan, 2017; Bayne and Carter, 2018; Pollan, 2018). For those involved in research with these compounds, it seems likely that they will influence psychology and psychiatry in a major way in the coming decades**—**but there is still much that is uncertain. In the present climate of rapid development, a compelling unified model of the brain mechanisms of psychedelics would hold significant value.

**Fig. 1.**
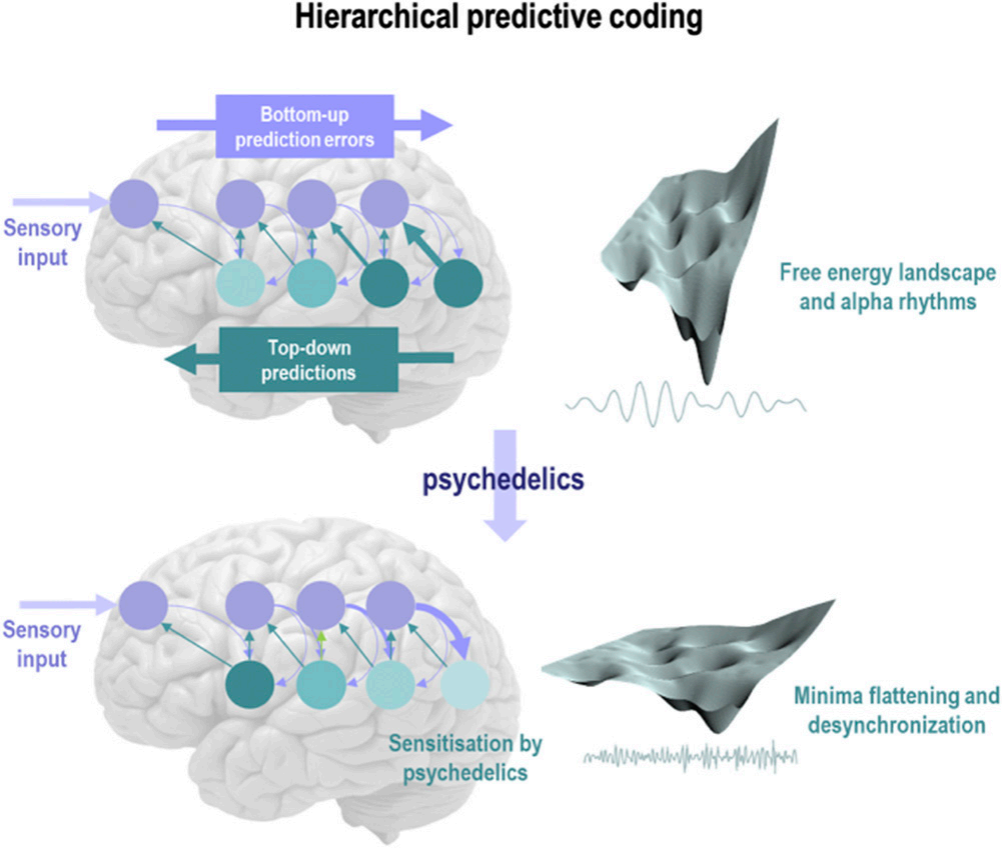
This schematic illustrates the proposed effects of psychedelics on hierarchical predictive coding, a predominant process theory for Bayesian inference and variational free-energy minimization in the brain. Under these computational architectures, sensory input arrives at the sensory epithelia and is compared with descending predictions. The ensuing prediction error (blue circles; e.g., neuronal populations of superficial pyramidal cells) is then passed forward into hierarchies, to update expectations at higher levels (blue arrows). These posterior expectations (teal circles; e.g., deep pyramidal cells) then generate predictions of the representations in lower levels, via descending predictions (teal arrows). The recurrent neuronal message passing (i.e., neuronal dynamics) tries to minimize the amplitude of prediction errors at each and every level of the hierarchy, thereby furnishing the best explanation for sensory input at multiple levels of hierarchal abstraction. Crucially, this process depends upon the precision (ascribed importance or salience) afforded to the ascending prediction errors (surprise) and the precision (felt confidence) of posterior beliefs. The basic idea—pursued in this article—is that psychedelics act preferentially via stimulating 5-HT2ARs on deep pyramidal cells within the visual cortex as well as at higher levels of the cortical hierarchy. Deep-layer pyramidal neurons are thought to encode posterior expectations, priors, or beliefs. The resulting disinhibition or sensitization of these units lightens to precision of higher-level expectations so that (by implication of the model) they are more sensitive to ascending prediction errors (surprise/ascending information), as indicated by the thick blue arrow in the lower panel. Computationally, this process corresponds to reducing the precision of higher-level prior beliefs and an implicit reduction in the curvature of the free-energy landscape that contains neuronal dynamics. Effectively, this can be thought of as a flattening of local minima, enabling neuronal dynamics to escape their basins of attraction and—when in flat minima—express long-range correlations and desynchronized activity. This schematic uses the format in Bastos et al. (2012), to which the reader is referred for details.

In this work, we propose such a unifying model. We call this relaxed beliefs under psychedelics (REBUS)^1^ and the anarchic brain or “REBUS” for short. The model takes inspiration from two formulations of brain function, namely: 1) the free-energy principle^2^ (Friston, 2010) and 2) the entropic brain hypothesis^3^ (Carhart-Harris, 2018a). The free-energy principle furnishes a unified description of the behavior of autopoietic or living (i.e., self-producing and maintaining) systems**—**that explains their development, processing, and behavior based on their inherent tendency to resist disorder and minimize uncertainty. This description of (self-evidencing) systems appeals to their inherent drive to optimize internal probabilistic representations**—**and sampling**—**of their environments (Friston, 2010). Hierarchical predictive coding forms a major part of the free-energy principle and, thus, the REBUS model also (see Fig. 1 and also the Supplemental Glossary for disambiguation of terms).

The entropic brain hypothesis proposes that within upper and lower bounds, i.e., a critical zone (Hilgetag and Hutt, 2014), the entropy of spontaneous brain activity indexes the richness (i.e., the diversity and vividness) of subjective experience, within any given state of consciousness, and that psychedelics acutely increase both (Carhart-Harris et al., 2014; Carhart-Harris, 2018a). Crucially, both the free-energy and entropic brain formulations rest on quantifiable measures from information theory that are apt for empirical study.

The entropic brain hypothesis and free-energy principle are inter-related, not least because of their shared appeal to information theoretical metrics, closely linked to classic (Shannon) entropy. In its most basic information-theoretical form, entropy is a dimensionless measure of uncertainty about a dynamical phenomenon (Ben-Naim, 2007). The entropic brain measures the uncertainty of neuronal fluctuations across time, whereas free-energy measures the uncertainty of beliefs encoded by neuronal fluctuations. The entropic brain hypothesis proposes that a principal action of psychedelics is to increase the entropy of spontaneous brain activity, and that such effects are mirrored at the subjective level by an increase in the richness of conscious experience, assuming that the brain and mind are flip sides of the same coin, i.e., a position commensurate with so-called “dual aspect monism” (Solms and Turnbull, 2003).

The entropic brain hypothesis began as a largely conceptual offering (Carhart-Harris et al., 2014) but has since been substantiated by a number of empirical neuroimaging studies and quantitative analyses (Carhart-Harris, 2018a). It is intended that the neurobiological evidence base for the entropic brain hypothesis be supplemented by concomitant measures of subjective and behavioral complexity/entropy**—**as have been applied in the context of development and ageing (Gauvrit et al., 2017) and natural language (Hoffman et al., 2013). Thus, the entropic mind and behavior are natural extensions of the entropic brain hypothesis.

In this study, we integrate the entropic brain hypothesis and the free-energy principle framework, and in particular, exploit the latter’s close links with predictive processing or coding and the so-called Bayesian brain hypothesis. Put simply, these closely related perspectives on brain and mind function maintain that the brain instantiates, within its hierarchical architecture, best-guess statistical approximations (generative models) of the causes of its sensorium based on Bayesian principles of empirically informed belief updating (Rao and Ballard, 1999). Predictive coding is a neuronally plausible theory for this belief updating**—**and has been used to account for a vast range of subjective and behavioral phenomena: from perceptual illusions in healthy individuals (Zeller et al., 2015) to a broad number of psychopathological states and disorders (Fletcher and Frith, 2009; Edwards et al., 2012). In fact, it is reasonable to say that hierarchical predictive coding now represents the predominant neurobiological and computational framework for describing psychological phenomena in health and disease.

The present work proposes that a principal action of classic psychedelics is to relax the precision weighting^4^ of prior beliefs encoded in the spontaneous activity of neuronal hierarchies. Statistically, precision is equivalent to inverse variance (i.e., negative entropy), and, subjectively and intuitively, it can be regarded as commensurate with felt confidence, i.e., the closer data are to a given model, the smaller will be our errors of prediction and thus, the stronger will be our felt confidence in our model. More specifically, this study proposes that the effect of this relaxation process is felt most profoundly when it occurs at the highest or deepest level of the brain’s functional architecture, i.e., the levels that instantiate particularly high-level models such as those related to selfhood, identity, or ego. Moreover, as outlined below, due to the distinct pharmacology of psychedelics**—**effects at high or deep levels of the brain’s functional hierarchy happen quite readily (perhaps definitively so) with this category of drug, particularly at high doses (Milliere, 2017).

Functionally, the effect of relaxing the precision weighting of high-level priors is to create a state in which these priors are imbued with less confidence. As just touched on, an important example of a high-level prior is the belief that one has a particular personality and set of characteristics and views. This (umbrella) belief is commensurate with the narrative self (Milliere, 2017) or ego (Carhart-Harris and Friston, 2010). It is proposed in this work that dissolving high-level priors has implications for the functioning of the rest of the hierarchy—and indeed the integrity of the hierarchy itself. More specifically, we propose that the general (entropic) action of psychedelics is to render the brain/mind’s (variational free) energy landscape flattened or opened up. It follows from hierarchical predictive coding that precise high-level priors or beliefs ordinarily have an important constraining influence on the rest of the hierarchy, canalizing lower components and inhibiting their expression and influence.

A corollary of relaxing high-level priors or beliefs under psychedelics is that ascending prediction errors from lower levels of the system (that are ordinarily unable to update beliefs due to the top-down suppressive influence of heavily-weighted priors) can find freer register in conscious experience, by reaching and impressing on higher levels of the hierarchy. In this work, we propose that this straightforward model can account for the full breadth of subjective phenomena associated with the psychedelic experience, including the following: ego dissolution (Nour et al., 2016; Milliere, 2017), the unitive and largely synonymous peak experience (Roseman et al., 2018b), near-death-like experiences (Timmermann et al., 2018), a sense of anxiety and uncertainty (Carhart-Harris et al., 2014), heightened suggestibility (Carhart-Harris et al., 2015), sensitivity to context (Carhart-Harris et al., 2018c), emotional lability (Carhart-Harris et al., 2016b), insight (Carbonaro et al., 2018), paranoid and delusional thinking (Carhart-Harris et al., 2016b), psychological age regression and vivid autobiographical recollection (Grof, 1979), recourse to magical thinking (Carhart-Harris et al., 2014), altered time perception (Wackermann et al., 2008), a sense of the ineffable (Pollan, 2018), entity encounters and sensed presence (Timmermann et al., 2018), eyes-closed dreamlike visions (de Araujo et al., 2012), geometric hallucinations (Bressloff et al., 2002), and more.

The hypothesized flattening of the brain’s (variational free) energy landscape^5^ under psychedelics can be seen as analogous to the phenomenon of simulated annealing in computer science**—**which itself is analogous to annealing in metallurgy, whereby a system is heated (i.e., instantiated by increased neural excitability), such that it attains a state of heightened plasticity, in which the discovery of new energy minima (relatively stable places/trajectories for the system to visit/reside in for a period of time) is accelerated (Wang and Smith, 1998). Subsequently, as the drug is metabolized and the system cools, its dynamics begin to stabilize**—**and attractor basins begin to steepen again (Carhart-Harris et al., 2017). This process may result in the emergence of a new energy landscape with revised properties (Fig. 1).

**Fig. 2.**
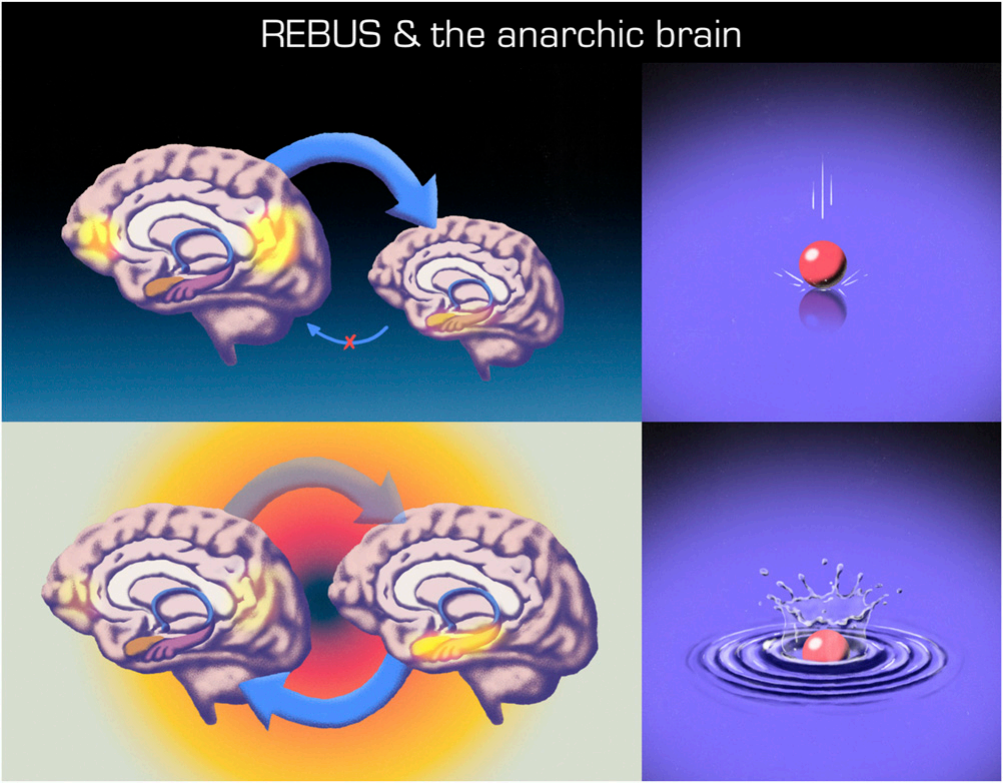
These schematics depict: on the top row, brain organization in psychopathologies such as depression in which high-level priors (e.g., instantiated by the DMN) are overweighted (thick top-down arrow), causing a suppression of and insensitivity to bottom-up signaling (e.g., stemming from the limbic system). In this figure, we show compromised bottom-up signaling via a thin arrow with a red cross over its center. The graphic on the top right depicts a pathologically rigid or frozen system, insensitive to perturbation, represented in this figure as a heavy ball dropped on a solid surface having a minimal effect on the system, i.e., the ball lands with an uneventful thud. The bottom row depicts brain organization under a psychedelic. In this figure, the top-down arrow has been made translucent to reflect a deweighting or relaxation of high-level priors or beliefs (this component of the model is referred to by the acronym REBUS). The effect of this deweighting is to enable bottom-up information intrinsic to the system, to travel up the hierarchy with greater latitude and compass. We refer to this component of the model as the anarchic brain. That the two brains on the bottom row are on the same level and of the same size is intended to reflect a generalized decrease in hierarchical constraints under the psychedelic. The graphic on the bottom right represents a phenomenon known as critical slowing, i.e., systems at criticality display maximal sensitivity to perturbation. In this figure, one can see ripples appearing after a heavy ball is dropped into a liquid surface, reflecting how, in this particular system, and unlike its frozen counterpart above, there will be a slow recovery to the same perturbation. Illustrations by Pedro Oliveira, courtesy of Favo Studio.

Within the transient hot state of a psychedelic experience, a flattened landscape implies that attracting brain states (and accompanying mind states) encoding beliefs are less stable and influential, implying that interstate transitions can occur more freely. Thus, rather than the mind and brain being constrained to a small number of gravitationally dominant attractors (i.e., states or sequence of states), the mind and brain spontaneously transition between states with greater freedom**—**and in a less predictable way. These altered dynamics may be felt as an enriched or broadened global state of consciousness**—**and a sense of the mind opening up (Atasoy et al., 2017). Equally, however, they may be felt as aversive and disconcerting (Carhart-Harris et al., 2016b), especially given that small perturbations to the system can have large repercussions in such a flattened energy landscape, e.g., see the related themes of context sensitivity (Carhart-Harris et al., 2018c) and critical slowing (Cocchi et al., 2017); see Fig. 2.

Under psychedelics, one can view global brain function as entering a mode or state that: 1) features a lightening or relaxation of the precision weighting on priors and 2) allows for a (potentially enduring) revision of such priors, via the release of prediction error that impacts on the sensitized priors. Empirically,^6^ findings of an enriched repertoire of connectivity motifs (Tagliazucchi et al., 2014) and connectome harmonics under psychedelics (Atasoy et al., 2017) may be seen as consistent with the notion of a flattened energy landscape under these drugs. We also go further to speculate that the influence of previously dominant attractors**—**including those that may be pathologic**—**perhaps erected in response to trauma-related uncertainty or ambiguity, whether acute or chronic (Carhart-Harris, 2019), may be relaxed in an enduring way after successful psychedelic therapy, potentially accounting for the so-called afterglow period (Majic et al., 2015) and beyond.

In brief, our proposal is that psychedelics disrupt functioning at a level of the system (sensitivity of deep-layer pyramidal neurons, power of low-frequency rhythms, and integrity of large-scale networks) that encodes the precision of priors, beliefs, or assumptions. At low doses, subjective effects may be felt most tangibly at the perceptual level and particularly within the visual domain [serotonin 2A receptor (5-HT2ARs) are richly expressed within the visual cortex], but at higher doses, effects will become more profound as the functioning of higher levels of the global hierarchy become significantly disrupted, potentially accounting for phenomena such as the dissolution of ego boundaries^7^ and potential (long-term) revision of high-level priors, perspectives, or beliefs.

Mechanisms of simulated annealing, the destruction of sharp minima, and Bayesian model reduction (BMR) can be raised in this work (and in more detail later on) to account for the rapid, marked, and enduring belief changes that can be seen after (high-dose) psychedelic experience (Friston et al., 2017; Lyons and Carhart-Harris, 2018a)**—**and upon which the growing evidence for psychedelic therapy is based (Carhart-Harris and Goodwin, 2017; Rucker et al., 2018). We propose that many, if not most, psychopathologies develop via the gradual (or rapid**—**in the case of acute trauma) entrenchment of pathologic thoughts and behaviors, plus aberrant beliefs held at a high level, e.g., in the form of negative self-perception and/or fearful, pessimistic, and sometimes paranoid outlooks. We also propose that these pathologic beliefs are ascribed excessive precision, weight, or influence in many psychiatric disorders (Clark et al., 2018). This view overlaps somewhat with the so-called capture theory of mental suffering (Kessler, 2016) as well as Bayesian perspectives on delusional belief formation in psychosis (Adams et al., 2018; Sterzer et al., 2018).

The process of capture (Kessler, 2016) or tightening of beliefs (and behaviors) in uncertainty (Carhart-Harris, 2019) may begin with psychological injury (Hovens et al., 2015; Peters et al., 2017)^8^ and then develop progressively, likely as an (functional) attempt to mitigate the averseness of felt uncertainty or ambiguity brought on by the relevant shock(s) and/or psychological injury or injuries. In this sense, the creation of the aberrant belief is self-protective and defensive (i.e., it is a coping mechanism), and, although pathologic, it may work to reduce an individual’s felt uncertainty. Indeed, as a strategy for suppressing uncertainty, even aberrant belief formation is consistent with the uncertainty-resolving imperatives of the free-energy principle. However, because the relevant defensive belief(s) becomes defining of pathology, it follows that it must be revised to avoid becoming entrenched and thus resistant to treatment. Moreover, because the belief(s) is self-protective, it is natural that psychological resistance will be felt if its integrity is threatened, e.g., pharmacologically via a psychedelic, or in the context of psychodynamic psychotherapy. It also follows that the precision weighting of the relevant high-level prior(s) or beliefs must first be relaxed, before they can be revised, and that a safe container for such a potentially destabilizing process is essential (Carhart-Harris et al., 2018c).

The ideal result of the process of belief relaxation and revision is a recalibration of the relevant beliefs so that they may better align or harmonize with other levels of the system and with bottom-up information—whether originating from within (e.g., via lower-level intrinsic systems and related interoception) or, at lower doses, outside the individual (i.e., via sensory input or extroception). Such functional harmony or realignment may look like a system better able to guide thought and behavior in an open, unguarded way (Watts et al., 2017; Carhart-Harris et al., 2018b). In what follows, we offer some speculations on how the proposed top heaviness underpinning much psychopathology^9^ is biologically instantiated in the human brain—and potentially relieved by psychedelic therapy.

This work will endeavor to account for a broad range of phenomena associated with the psychedelic experience by first presenting the neurobiological case for the hypothesis that the precision weighting of beliefs is relaxed under these compounds (REBUS). We then focus on the phenomenology of the psychedelic experience and its consequences—and how this ties in with the relevant neurobiology. We hope to explain how a potential relaxation of the precision weighting of high-level priors or beliefs and subsequent opening up of mental content under a psychedelic is logically implied by our two main guiding formulations: the entropic brain hypothesis and the free-energy principle. An important complementary theme, inspired by the so-called algorithmic information theory account of consciousness (Ruffini, 2017), is that the brain’s highest levels naturally envelope the content of levels below, thereby effectively suppressing their potential information content via the action of a summary model.^10^ Such models are referred to as compressive because they compress the content of levels below, effectively causing their content to disappear or at least not be heard. By implication, the breakdown of a compressive model should have an expansive influence on the system’s potential information content, as it would liberate suppressed information, enabling it to travel up through the hierarchy, impressing on higher levels as it does.

Our empirically informed theoretical treatment of psychedelics begins with a review of their pharmacology and associated behavioral research, both in animals and humans, making special reference to synaptic efficacy-enhancing effects (Ly et al., 2018). We then address the important relationship between their key pharmacological action, namely, 5-HT2AR agonism and sensitivity to context (Carhart-Harris et al., 2018c). We place special emphasis on the interaction between perceived (or actual) environmental volatility, associated stress, upregulation of 5-HT2AR functioning, and associated plasticity.

We argue that this upregulation of 5-HT2AR functioning facilitates adaptability to environmental volatility (Carhart-Harris and Nutt, 2017). This property has also been attributed to other neuromodulatory systems, such as noradrenaline and acetylcholine (Yu and Dayan, 2002, 2005), but it may be an especially pronounced feature of the 5-HT2AR system (Carhart-Harris and Nutt, 2017). We then turn to functional brain-imaging studies with psychedelics and what these tell us about the acute action of psychedelics and their potential longer-term effects. We conclude by focusing on recent evidence supporting the therapeutic potential of psychedelics and offer a unified account of their acute and therapeutic mechanisms based on an integration of the entropic brain hypothesis and free-energy principle.

## II. The REBUS Model

### A. The Basic Neuropharmacology of Psychedelics

Some classic psychedelics, such as LSD, have a rich pharmacology (Nichols, 2004, 2016); however, there is compelling evidence that activation of a specific serotonin receptor subtype, the 5-HT2AR, is necessary for these compounds’ subsequent psychedelic effects. The affinity of psychedelics for the 5-HT2AR correlates strongly with their subjective and behavioral potency in humans and animals (Glennon et al., 1992), and pretreatment with 5-HT2AR antagonists markedly attenuates their characteristic psychological effects in humans (Vollenweider et al., 1998).^11^ The 5-HT2ARs are expressed most densely in the cortex and especially in high-level association regions, such as those belonging to the so-called default-mode network (DMN) (Beliveau et al., 2017). Recently, it was found that 5-HT2AR occupancy and plasma concentration of psilocin (the active metabolite of psilocybin) correlate with psilocybin’s subjective effects—in a nonlinear way that matches the nonlinear nature of psychological phenomena such as ego dissolution (Madsen et al., 2019).

The DMN is a high-level brain network that is exceptional for a number of reasons, including: 1) its high basal metabolism (Raichle, 2015), 2) dense interregional connectivity (Hagmann et al., 2008), 3) extreme heteromodality (Margulies et al., 2016), and 4) association with functionally high-level behaviors—falling broadly under the constructs of perspective taking and metacognition (Soto et al., 2018). The DMN has been associated with arguably species-defining behaviors such as mental time travel (Ostby et al., 2012), moral decision making (Kaplan et al., 2017), counter-factual thinking (Van Hoeck et al., 2013), and self-consciousness (Fingelkurts et al., 2012). Some years ago, we nominated the DMN as the primary biologic substrate of the Freudian ego (Carhart-Harris and Friston, 2010), as well as a vital system for the maintenance of secondary process cognition (Carhart-Harris et al., 2014). With some qualifications (Lebedev et al., 2015) and an acknowledgment of the looseness of this attributional exercise (mainly due to the intrinsic abstraction of the constructs in question, which is a challenge to surmount), we still largely maintain these views (see also Davey et al., 2016; Davey and Harrison, 2018). In simpler, more mechanistic terms, however, the human DMN can be considered to sit at the top end—or center—of a uniquely deep hierarchical system, i.e., the human brain, instantiating a uniquely deep and domain-general model of the embodied agent his- or herself (Friston, 2018; Friston et al., 2018). See Becker (2011) for a reminder of the unique depth of human mental models and their psychological and sociological implications.

An important aspect of the present thesis is that the potential belief-revising effects of psychedelics occur via an action on the top end (or deepest aspect) of the brain’s functional architecture, with high-level cortex being particularly implicated. This high-end locus of action is supported by the especially dense expression of 5-HT2ARs within high-level cortex (Beliveau et al., 2017). Functional disintegration of the DMN^12^ and changes in other high-level networks (Lebedev et al., 2015; Tagliazucchi et al., 2016) have been linked with the most abstract phenomenological features of the psychedelic experience, such as ego dissolution (Carhart-Harris et al., 2016c); however, we also propose that the pronounced perceptual effects of psychedelics involve effects both within and above the relevant sensory modules. Consistent with the free-energy principle, we propose that the brain’s highest levels provide an implicit, centralizing, and generalized compression of (potential) information held within and processed by subordinate levels; i.e., the high or deep hierarchical levels furnish beliefs about abstract, domain-general narratives regarding the world qualities of self and states of being. Moreover, these beliefs also offer the best explanation for inferences at lower levels (Ruffini, 2017), a rule that also applies to the extensive architecture dedicated to perceptual inference (Gregory, 1980). Relatedly, although the DMN is characteristically far removed from immediate sensory processing (Margulies et al., 2016), evidence suggests that it contextualizes sensory processing, particularly in the visual modality (Huang and Sereno, 2013; Griffis et al., 2017), suggesting there is little that is beyond the reach of the DMN (even if its influence is somewhat detached), which is what one would expect from a particularly high-level component of a system, i.e., an exceptionally broad executive reach or influence.

It is hypothesized that disrupting the broad compressive function of the DMN and related high-level networks has trickle-down effects on the functional organization of the global system. In addition to the brain’s highest hierarchical levels, we highlight how intermediate hierarchical levels (i.e., those concerned with cognition, perception, and emotion) are likely to be affected by psychedelics. Although this rule may be true for much of the brain, the operation of its most rudimentary, domain-specific levels (e.g., those concerned with motor and autonomic functions) may remain largely immune from the influence of psychedelics—at least on an implementation level. For example, under the effects of a high dose of a psychedelic, an individual may report feeling as if they cannot move, vocalize, or even breathe properly, but, in practice, they can typically perform these functions almost as normal.

#### 1. Interim Summary

Consistent with hierarchical predictive processing, we maintain that the highest level of the brain’s functional architecture ordinarily exerts an important constraining and compressing influence on perception, cognition, and emotion, so that perceptual anomalies and ambiguities—as well as dissonance and incongruence—are easily and effortlessly explained away via the invocation of broad, domain-general compressive narratives. In this work, we suggest that psychedelics impair this compressive function, resulting in a decompression of the mind-at-large—and that this is their most definitive mind-manifesting action.

One cannot discount the possibility that a direct disruption of priors instantiated by lower levels of the functional hierarchy occurs with psychedelics, and, indeed, an intermediate-level locus of action is supported by the predominant expression of 5-HT2ARs on layer 5 pyramidal neurons throughout much of the cortex, e.g., with particularly high expression within the primary visual cortex (Beliveau et al., 2017). This said, we still maintain that the especially high expression of 5-HT2ARs within high-level cortex is likely to be important. Moreover, this hierarchical view is consistent with the functional (hierarchical and recurrent message passing) asymmetries on which predictive coding depends. In short, although an intermediate action of psychedelics is plausible—if not likely—dysregulation of the highest hierarchical level may be the definitive action of psychedelics. See Pink-Hashkes et al. (2017) for discussion of a related and largely consistent hierarchical predictive-processing–based model of the action of psychedelics.

To illustrate this point, consider the example of hallucinated motion, e.g., perceiving motion in scenes that are actually static, such as seeing walls breathing, a classic experience with moderate doses of psychedelics. This phenomenon can be fairly viewed as relatively low level, i.e., as an anomaly of visual perception. However, we propose that its basis in the brain is not necessarily entirely low-level but may also arise due to an inability of high-level cortex to effectively constrain the relevant lower levels of the (visual) system. These levels include cortical regions that send information to V5, the motion-sensitive module of the visual system. Ordinarily, the assumption “walls don’t breathe” is so heavily weighted that it is rendered implicit (and therefore effectively silent) by a confident (highly-weighted) summarizing prior or compressive model. However, under a psychedelic, V5 may be forced to interpret increased signaling arising from lower-level units because of a functional negligence, not just within V5 itself, but also higher up in the hierarchy. Findings of impaired high- but not low-level motion perception with psilocybin could be interpreted as broadly consistent with this model, namely, pinning the main source of disruption high up in the brain’s functional hierarchy (Carter et al., 2004). The exceptionally high expression of 5-HT2ARs in the brain’s highest hierarchical levels provides a solid anatomic basis for this assumption, although we recognize expression is also particularly high in the primary visual cortex (Beliveau et al., 2017). Finally, the predominantly high-level expression of 5-HT2ARs might also explain the phenomenon of microdosing, namely, the taking of subsensory-perceptible doses of psychedelics that nevertheless (allegedly) produce a discernible cognitive/emotional effect (Waldman, 2017)—presumably again, through a (subtle) flattening of the brain’s functional hierarchy and associated (subtle) increase in bottom-up signaling.

### B. Rhythms, Networks, and the Relaxation of High-Level Priors

Activation of 5-HT2ARs induces an increase in excitatory postsynaptic currents and discharge rates in pyramidal neurons (Celada et al., 2008), causing an asynchronous mode of glutamate release (Aghajanian and Marek, 1999), also known as recurrent network activity (Aghajanian, 2009), and temporal dissociation between cortical pyramidal cell firing and the active phase of local field potentials, i.e., a spike-field decoherence (Celada et al., 2008). This irregular excitation of deep-layer pyramidal neurons is likely to account for the highly reliable finding of psychedelic-induced decreases in oscillatory activity (particularly in its most prominent frequency bands, such as alpha) in animal and human cortex (Muthukumaraswamy et al., 2013; Riga et al., 2014), a view that is substantiated by our application of dynamic causal modeling to psilocybin magnetoencephalography (MEG) data, which implicated layer 5 pyramidal neuron excitation as the cause of broadband decreases in oscillatory power (Muthukumaraswamy et al., 2013). From the perspective of predictive coding, these selective effects on deep pyramidal cells are particularly prescient because these populations are thought to encode expectations about the latent or hidden causes of the sensorium. Rendering them selectively more sensitive to ascending input (e.g., prediction errors) is precisely the neuromodulatory change that reduces the precision weighting of the expectations they encode. In other words, being more excitable is the physiologic mechanism that destroys sharp minima and underwrites a weakening of prior expectations.

It is well known that the brain’s resting power spectrum exhibits stark nonuniformities in its oscillatory components, in the sense that certain rhythms are especially pronounced. This seems to be particularly the case when recording population-level activity in the cortex. Perhaps the most conspicuous example of a predominant rhythm is the *α* rhythm or Berger wave of about 10 Hz, which shows a striking prominence, especially, but by no means exclusively, during eyes-closed waking rest. The *α* rhythm has been associated with a range of functions, including top-down inhibition (Klimesch et al., 2007). Evidence has been accumulating, however, that *α* also plays a more specific role in conferring top-down expectations about perceptual stimuli (Mayer et al., 2016) that effectively silence more granular information processed by lower-level aspects of the system. The *α* power is known to correlate positively with DMN activity (Mantini et al., 2007), as does *β* (Mantini et al., 2007). Like alpha, the *β* rhythm has also been associated with a top-down function, albeit within the motor system (Fries, 2015).

Another interpretation of *α* is that it is associated with a resting, reflective brain that responds—electrophysiologically speaking—with the emergence of higher frequencies on stimulation. This is known as event-related desynchronization (Pfurtscheller et al., 1996; Singh et al., 2002) and typically involves a shift to broadband activity. In brief, high *α* activity would appear to correspond to a neurophysiological state of low entropy and strong or precise (i.e., heavily-weighted) top-down constraints—as maintained by the free-energy principle (Boly et al., 2012; Bauer et al., 2014; Bastos et al., 2015).

Crucially, psychedelics have been found to dramatically decrease prominent low-frequency and therefore high-level brain rhythms such as *α* and *β*, and this effect appears to be both reliable and closely related to the intensity of their subjective effects (Muthukumaraswamy et al., 2013; Carhart-Harris et al., 2016c). Perhaps relatedly, psychedelics have also been found to tune brain activity closer to criticality (Atasoy et al., 2017; Muthukumaraswamy and Liley, 2018; Varley et al., 2019), such that it displays more of a scale-free organization (Muthukumaraswamy and Liley, 2018), an effect that may be mediated by *α* modulation (Muthukumaraswamy and Liley, 2018).

Using MEG, we found reduced *α* power in the posterior cingulate cortex under psychedelics, a major node of the DMN, and this effect correlated significantly with subjective ratings of ego dissolution under both psilocybin (Muthukumaraswamy et al., 2013) and LSD (Carhart-Harris et al., 2016c). The *α* rhythm is known to be especially highly expressed in humans relative to other animals, and especially so during maturity, with a peak at about 20 years of age (Basar and Guntekin, 2009), which, intriguingly, is approximately when the complexity of cognition has been found to be maximal (Gauvrit et al., 2017). Although open to critique, a curious cross-cultural electroencephalogram (EEG) study sampling eyes-closed brain activity in more than 400 individuals has reported that *α* is most pronounced in the most technologically advanced and developed cultures (Parameshwaran and Thiagarajan, 2017).

#### 1. Interim Summary

To summarize, evidence that psychedelics have a particular action on high-level cellular, oscillatory, and regional/network features of the brain’s functional architecture, combined with evidence that these features are implicated in a top-down predictive function, converges on a key hypothesis of this work, namely, that psychedelics have an important dysregulatory effect on the highest levels of the brain, subverting the brain’s ability to entrain and constrain emotion and perception to a central narrative:

“*The Centre cannot hold*.” (Yeats, 1865–1939)

We maintain that the hierarchically high-level character of key sites of action of psychedelics, e.g., 5-HT2ARs on deep-layer pyramidal neurons in high-level cortex/systems, is important, and that activation of these receptors renders the high-level components on which they sit, sensitized and functionally dysregulated. We propose that this dysregulation of the highest levels of the brain’s functional hierarchy is commensurate with a lightening of the precision weighting encoded by these levels and their associated dynamics. The effect of this, we propose, is to lighten the top heaviness of human cognition, by (temporarily) flattening the hierarchical organization that supports it. Although speculative, the alleged positive mood and procognitive effects of microdosing may depend on the same (temporary) antihierarchical effect, liberating brain and mind function, albeit with far greater subtlety than with higher doses.

At the other extreme, the sense of losing one’s self or ego with higher doses of psychedelics may explain why one can feel as if one is dying while under the effects of these drugs (Timmermann et al., 2018), despite the fact that peripheral physiology remains largely unaffected. Living systems adhere to the free-energy principle but dying systems do not (Friston, 2018). Indeed, a dying or dead brain/body is the very epitome of an entropic brain/body. The so-called entropic brain effect (Carhart-Harris, 2018a), exemplified by findings of increased brain complexity/entropy (Schartner et al., 2017) and decreased modular differentiation (Petri et al., 2014), provides an iconic image of dysregulated global brain function under psychedelics, with the 5-HT2AR as the trigger point.

### C. Plasticity and the Dissolution of (High-Level) Priors under Psychedelics

In this section, we address neurobiological and behavioral evidence for the hypothesis that psychedelics work by weakening the precision weighting of high-level priors and, thus, their ability to exert hierarchical control over and be impervious to the influence of lower-level components of the brain. Readers will note that psychedelics appear to affect predictive processing at a broad range of functional levels. It is our view that psychedelics affect not just high-level priors but intermediate-level priors also (e.g., those instantiated within the visual cortex). However, we maintain that the relaxation of precision on the high hierarchical levels has the most dramatic psychological consequences. One reason for this may be that these high levels encode cognition at a level of abstraction that is more removed from the (anchoring and entraining influence of) statistical regularities within the sensorium—and so are more able to change or undergo revision. Another reason why disruption at the highest levels may have special implications is that these high levels support domain-nonspecific narratives that influence the entire hierarchy. The principle is that the influence of the high levels runs deep, such that affecting them has particularly large or general implications.

Returning to a lower level, a recent study used optogenetics in mice to selectively measure the activity of serotonergic cells within the dorsal raphe nuclei (Matias et al., 2017). The authors found that dorsal raphe nuclei cell firing increased significantly under conditions of uncertainty, being sensitive to surprising events irrespective of their reward value (i.e., value-nonspecific prediction error). The authors inferred from these results that serotonin transmission plays an important role in enhancing adaptability in the face of uncertainty, a theme that chimes well with the model we are presenting in this work, as well as another presented recently (Carhart-Harris and Nutt, 2017), which specifically associates 5-HT2AR signaling with enhanced adaptability, particularly in situations of crisis. Thus, understanding the REBUS/anarchic brain model may have important implications for our understanding of brain serotonin function (Carhart-Harris and Nutt, 2017), a notable enigma in psychopharmacology.

The role of serotonin (Matias et al., 2017) and, more specifically, 5-HT2AR signaling (Boulougouris et al., 2008), in cognitive flexibility, has been substantiated by numerous studies in animals and humans, e.g., see Carhart-Harris and Nutt (2017) for a review. Psychedelics have been found to promote divergent thinking (Kuypers et al., 2016)—a key component of creative thinking—as well as an expansion of associative processing (Spitzer et al., 1996), while (unsurprisingly) impairing conventional cognition (Bayne and Carter, 2018), including convergent/discriminatory cognition (Kuypers et al., 2016). Recent (Berthoux et al., 2018; Ly et al., 2018) and older work (Vaidya et al., 1997) provide convincing evidence that 5-HT2AR signaling can enhance neural plasticity, as well as low-level learning and extinction learning, as reviewed in this study (Carhart-Harris and Nutt, 2017), suggesting that psychedelics promote a generalized plasticity via their agonist actions at the 5-HT2AR (Carhart-Harris et al., 2016b; Carhart-Harris and Nutt, 2017).

Serotonin transmission is known to play an important role in normal brain development (Azmitia, 2001; Maya Vetencourt et al., 2008) and has been increasingly linked to enhanced sensitivity to environmental influences (Branchi, 2011; Alboni et al., 2017). Relatedly, psychedelics appear to induce a regressive state, both behaviorally (Grof, 1979) and in terms of brain function (Roseman et al., 2014; Carhart-Harris et al., 2016c). Moreover, as in infancy, the influence of context has an exaggerated role under psychedelics (Carhart-Harris et al., 2018c), a characteristic property of critical systems^13^ linked to critical slowing^14^ (see section *II.F.3. Criticality and Optimality* for further discussion of the theme of criticality). It is also worth noting that 5-HT2AR densities are highest early in life, dropping significantly when adulthood is reached (Sheline et al., 2002). Behavioral complexity also decreases at a similar age, after peaking at about 25 years of age (Gauvrit et al., 2017). Lastly, that both children and individuals on psychedelics are hypersuggestible (Nicolas et al., 2011; Carhart-Harris et al., 2015) is consistent with the principle that brain activity is tuned closer to criticality in these—likely related—brain states.

Taken together, the above-cited findings speak to the notion that the brain enters an entropic hot state under psychedelics in which synaptic efficacy and plasticity are elevated (Ly et al., 2018). The result of this window of exceptionally high plasticity may be to leave a legacy of potentially enduring functional and perhaps anatomic change (Ly et al., 2018). As the acute drug effects begin to subside, the system (brain) will settle back into its default regimen of efficient free-energy minimization, mirrored by a renewed subjective sense of familiarity and assuredness, but may not return entirely as before.

Recent functional imaging work with psychedelics suggests that large-scale intrinsic brain networks, such as the DMN, do indeed reintegrate after a psychedelic experience (Carhart-Harris et al., 2017). It is tempting to speculate that profound psychedelic experiences have a residual impact on the DMN, perhaps pruning some of its (redundant) parameters, in a manner that is consistent with synaptic homoeostasis and BMR (Tononi and Cirelli, 2006; Friston et al., 2017). Psychedelics may also lighten the precision weighting of DMN-related high-level priors in an enduring way. Further work is needed to test these hypotheses; however, we anticipate that long-term changes in DMN functioning may account for common reports of a lighter, freer state of mind after intense psychedelic experiences (Watts et al., 2017), particularly in the context of psychedelic therapy. This postacute period of well-being is often referred to as the psychedelic afterglow (Sampedro et al., 2017) and has been described in various ways: “I felt free, carefree, re-energized” (Watts et al., 2017); “The concrete coat had come off” (Watts et al., 2017); “All that day and well into the next, a high pressure system of well-being dominated my psychological weather” (Pollan, 2018).^15^

### D. Behavioral Evidence of Relaxed Priors under Psychedelics

The relaxation of the precision weighting of high-level priors under psychedelics is a central theme of the REBUS model. But what behavioral evidence is there for the implicit dissolution of prior beliefs? Although not directly concerned with high-level priors,^16^ one place we may find some relevant data is perceptual processing and, specifically, oddball paradigms such as the mismatch negativity, which directly engages predictive coding mechanisms (Garrido et al., 2009), and the engagement of lower-level priors and sensory prediction error. Using auditory mismatch negativity during MEG scanning, a blunted surprise response to deviant tones was found under LSD relative to placebo, as was reduced neuronal adaptation (Timmermann et al., 2017). This result makes sense based on the principle that psychedelics weaken expectations about standard tones in this paradigm, thus making deviant tones seem less anomalous or surprising. Using dynamical causal modeling, it was apparent that the diminished surprise signals could be best accounted for by reduced top-down information flow from the frontal cortex (Timmermann et al., 2017)—again consistent with the REBUS model of relaxed priors under psychedelics. Also consistent are findings from a study that measured visual evoked responses to Kanisza triangles, a visual perceptual illusion requiring object completion via top-down perceptual priors. Results revealed reduced object completion and related evoked potentials under psilocybin (Kometer et al., 2011), and these effects correlated with the intensity of spontaneous visual hallucinations. Another visual perceptual phenomenon that probes predictive coding is binocular rivalry, in which the presentation of two different visual stimuli to each eye typically induces the alternation between one percept and another. Under psilocybin, participants were more likely to perceive mixed percepts, and switch between them, consistent with reduced perceptual constraints, presumably due to impaired top-down resolution of perceptual uncertainty (Carter et al., 2005, 2007). A number of other studies have demonstrated reduced prepulse inhibition under psychedelics (Quednow et al., 2012; Schmid et al., 2015) and a 5-HT2AR–mediated augmentation of the startle response (Jiang et al., 2011), consistent with reduced top-down sensory inhibition or gating (Vollenweider and Geyer, 2001). Somewhat relatedly, we have seen increased information flow from the parahippocampus to the visual cortex during LSD-enhanced music listening (Kaelen et al., 2016), a phenomenon that can be linked to the hypothesized sensitization of cortex to bottom-up signaling under psychedelics, in this case stemming from a lower-level intrinsic system, i.e., the limbic system. It is noteworthy that the limbic system has been particularly implicated in the action of psychedelics (Carhart-Harris et al., 2014; Tagliazucchi et al., 2014; Lebedev et al., 2015).

An interesting study that directly addressed differential levels of perceptual processing under psilocybin found impairments in high- but not low-level motion perception (Carter et al., 2004). This finding makes sense given that prestimulus *α* oscillations have been found to assist high-level motion perception (Mayer et al., 2016) and *α* is known to be tonically reduced under psychedelics (Carhart-Harris et al., 2016c). The foregoing findings (Carter et al., 2004) could be interpreted as consistent with the notion that low-level autonomic functioning, which relies less on the invocation of complex internal models, is largely preserved under psychedelics, e.g., motor action is largely preserved, presumably because there is relatively low 5-HT2AR expression in motor cortex (Beliveau et al., 2017). Moreover, this may explain why low-level processing and indeed low-level learning (including extinction learning) appear to be not just preserved but enhanced with increased 5-HT2AR stimulation (King et al., 1972; Welsh et al., 1998; Harvey, 2003; Harvey et al., 2004; Romano et al., 2006, 2010; Gresch et al., 2007; Zhang et al., 2013), whereas conventional higher-level cognition is unsurprisingly impaired (Bayne and Carter, 2018).

Additional evidence for reduced top-down processing under psychedelics can be seen in changes in spontaneous brain activity, including the aforementioned reductions in the integrity of large-scale intrinsic networks (Carhart-Harris et al., 2016c), as well as the relative collapse of prominent oscillatory rhythms in the brain (Carhart-Harris et al., 2016c) that have been linked to these high-level networks (Laufs et al., 2003; Mantini et al., 2007). Additionally, decreased top-down information flow has been found when transfer entropy was applied to EEG data recorded under ayahuasca (Alonso et al., 2015) and when dynamic causal modeling was applied to resting-state functional magnetic resonance imaging data, suggesting reduced top-down flow from the posterior cingulate cortex to the thalamus (Preller et al., 2019). The potentially related finding of increased thalamic–cortical information flow in this study (Preller et al., 2019) also ties in with others (Muller et al., 2017) that have proposed (Geyer and Vollenweider, 2008) and demonstrated (Halberstadt and Geyer, 2018) reduced sensory gating with psychedelics. Increased flow of sensory information up the brain’s functional hierarchy is consistent with the general hypothesis proposed in this work that relaxed high-level priors (REBUS) occur in parallel with increased bottom-up signaling (anarchic brain)—as the two model components are mutually dependent. Indeed, this intimate relationship between priors and prediction error is an integral part of the hierarchical prediction-processing model.

Psychedelics have, of course, also been found to affect higher level aspects of information processing, such as emotional (Kaelen et al., 2015) and social processing (Preller et al., 2016), mental time travel (Speth et al., 2016), imagination (Carhart-Harris et al., 2012b), and ego functioning in general (Nour et al., 2016; Milliere, 2017). We propose that the action of psychedelics on the precision weighting of high-level priors is (one-half of) their definitive psychological action (the other half being increased bottom-up signaling, hence the anarchic brain component). With sufficiently high doses, time and self are reported to lose meaning (Turton et al., 2014); emotions show greater lability (Carhart-Harris et al., 2016b), but can also be felt with greater depth and meaning (Preller et al., 2017); and a paradoxical intermixing of mood states can occur (Carhart-Harris et al., 2016b). Emotional responsiveness to conventional experimental stimuli, such as series of static facial expressions, appears to be reduced (Kraehenmann et al., 2015a), accompanied by reduced top-down connectivity (Kraehenmann et al., 2015b). However, this may relate to a generalized disengagement with conventional behavioral paradigms, which is a particular problem in psychedelic research. See Carhart-Harris (2018a) for a discussion of this matter.

As noted above and consistent with a relaxation of high-level priors, social processing appears to be affected under psychedelics. For example, reduced feelings of social exclusion were reported (under psilocybin) in a behavioral paradigm designed to induce relevant feelings of exclusion (Preller et al., 2016), and increased emotional empathy was seen in a separate paradigm (Pokorny et al., 2017). Reduced rejection of unfair monetary offers has been observed under psilocybin and *3,4-methylenedioxy-methamphetamine* (MDMA), whereas an additional increase in generosity was seen with MDMA (Gabay et al., 2018). The authors interpret these results as consistent with a prosocial effect, something that appears to be especially characteristic of the MDMA experience (Bedi et al., 2014). MDMA is not a classic psychedelic, but, like psilocybin (Kometer et al., 2012), its positive mood effects have been linked to increased 5-HT2AR activity (van Wel et al., 2012). Although this may be so, it is essential to emphasize that MDMA is not a direct 5-HT2AR agonist; rather, MDMA is likely to engage all serotonin receptors indirectly through its potent release of serotonin (Bradbury et al., 2014). We speculate that MDMA’s prosocial effects, including its positive effects on social approach (Kamilar-Britt and Bedi, 2015), may relate (at least in part) to raised activity at 5-HT2ARs (via increased endogenous serotonin release), causing a relaxation of socially relevant assumptions (beliefs) that ordinarily inhibit social approach. This effect, combined with a reduction of anxiety via serotonin’s action at its other receptors (e.g., postsynaptic serotonin 1A receptors in limbic circuitry) and an increase in arousal and confidence via its release of noradrenaline and dopamine, is likely to account for the characteristic subjective effects of MDMA; see Carhart-Harris and Nutt (2017) for a related discussion.

### E. Comparisons with Other Altered States and Traits

#### 1. Psychosis

It is natural to ask how the hypothesized changes in brain and mind function under classic psychedelics relate to other, perhaps scientifically and medically more familiar, and thus tangible, states and traits. The psychedelic state has traditionally been compared with psychosis, but this comparison needs to be carefully qualified (Carhart-Harris et al., 2016b). It is well known that there is considerable heterogeneity to psychosis. For example, within the same individual, periods of (near) remission can bookend acute psychotic episodes featuring florid symptomatology. Moreover, whereas one person may experience florid positive symptoms associated with his/her first psychotic episode, another may present with a complex, but stable delusional framework coexistent with predominantly negative symptoms.

It has previously been suggested and demonstrated (Gouzoulis et al., 1994; Carhart-Harris et al., 2016b) that the acute psychedelic experience is a much better model of the early (and perhaps acute) symptoms of an incipient psychosis than symptoms associated with chronic schizophrenia, presumably because the underlying brain states of early psychosis (but not developed) schizophrenia share similarities with those underlying the psychedelic state. We speculate that reduced precision weighting on high-level priors may underlie these commonalities. This speculation could explain common phenomenological features of early psychosis and the psychedelic state, such as a fragmented sense of self and a basal anxious uncertainty that, if sufficiently persistent and intolerable, may be brought under control through the formation of an overarching delusional belief system. See Carhart-Harris and Nutt (2017) for a relevant discussion. This schema of weak high-level priors concomitant with heavily weighted (i.e., precise) prediction errors, manifesting as aberrant salience (Kapur, 2003) in the early phase of a psychosis, followed by the formation of heavily weighted and therefore stabilizing, but delusional high-level priors, has been discussed before (Clark, 2016).

We posit that the key difference between the psychedelic state and schizophrenia is that a delusional belief system does not (typically) crystallize in the psychedelic state for the following reasons: 1) unlike in psychosis, in the psychedelic state, the system (typically) begins from a baseline state of stable high-level priors (e.g., a stable ego) to which it returns as drug effects subside; 2) drug effects typically subside (e.g., after about 3–5 hours in the case of psilocybin); thus, there is insufficient opportunity or need for a delusional belief system to close out uncertainty; 3) increased prediction error (and accompanying increased uncertainty) in the psychedelic state is typically seen as an acceptable, expected, and even valued part of the drug experience (see section *II.F.Psychedelics and Insight* on insight below) and thus is typically learned from, i.e., integrated rather defended against, possibly via the formation of delusional beliefs and flight from reality (as in schizophrenia); and 4) there is no evidence that prediction errors are overweighted in the psychedelic state; i.e., aberrant salient is not the rule.

Using Pavlovian conditioning, an impressive recent study entrained the acquisition of stimulus-driven auditory hallucinations in a range of participant groups (Powers et al., 2017). This effect was linked to endogenously occurring hallucinations in pathologic conditions such as schizophrenia. This so-called strong priors model of hallucinations and delusions is relatively appealing (Sterzer et al., 2018). Some might be tempted to extrapolate from the above study’s findings to either perceptual abnormalities in the psychedelic state or indeed early psychosis. However, our position is that early psychosis and the psychedelic stare do not fit the strong priors model well for the reasons discussed above.

In brief, our view of the etiology of schizophrenia is that, for whatever combination of developmental, environmental, and/or genetic reasons, within certain at-risk individuals, the brain’s highest-level priors are ineffective, meaning bottom-up prediction errors travel more freely up the hierarchy, affecting it as it does.^17^ This much is consistent with the psychedelic experience. However, in a developing psychosis, we assume that the cycle of weak priors, high prediction error is more sustained than in the psychedelic experience. This, combined with adverse contextual conditions (Zammit et al., 2010), may contribute to ascending signals being ascribed greater precision in an emergent psychosis, e.g., due to the recruitment of dopaminergic mechanisms (Pehek et al., 2006; Pehek and Hernan, 2015), manifesting in the classic phenomenon of aberrant salience (Kapur, 2003). These critical secondary factors that are not by default part of the psychedelic picture most likely serve to encourage (delusional) interpretation of the (amped-up) prediction errors in an effort to reduce free energy and thus subdue and stabilize the system.

#### 2. Hallucinogen-Persisting Perceptual Disorder

Hallucinogen-persisting perceptual disorder (HPPD) is a Diagnostic and Statistical Manual of Mental Disorders, 5th Edition–listed disorder that relates to enduring visual perceptual abnormalities that persist beyond an acute psychedelic drug experience. Its prevalence appears to be low and its etiology complex, but symptoms can still be distressing for individuals (Halpern et al., 2018). Under the REBUS model, it is natural to speculate that HPPD may occur if/when the collapse of hierarchical message passing does not fully recover. A compromised hierarchy would imply a compromised suppression of prediction error, and it is natural to assume that persistent perceptual abnormalities reflect attempts to explain away irreducible prediction errors. Future brain-imaging work could examine whether aspects of hierarchical message passing, such as top-down effective connectivity, are indeed compromised in individuals reporting HPPD.

#### 3. Autism

Autism is another disorder in which aberrant high-level priors have been thought to play a role (Van de Cruys et al., 2014; van Schalkwyk et al., 2017). On first glance, the (hypothesized) mechanics of autism may appear to share similarities with those proposed in this work to underlie the psychedelic state, i.e., aberrant precision weighting on high-level priors (e.g., relevant to social processing). Added to this, in autism, there appears to be a compensatory upregulation of attention to and thus precision weighting of lower-level priors and sensory input. Consistently, in autism, imprecise and/or rigid high-level priors have been linked to deficient self-reflectiveness and theory of mind, as well as a biasing of attention toward the more elemental and domain-specific features of the sensorium (Van de Cruys et al., 2014). Relatedly, a reduction in perceptual grouping has been observed in autism (Goris et al., 2018), as it has in the psychedelic state (Kometer et al., 2011). It is easy to intuit some important differences in the mechanics of autism and the psychedelic state, however, not least because the phenomenology appears so different. For example, people show increased interest in others under psychedelics and an enhanced awareness of emotion, self-schemas, and interpersonal relationships (Preller et al., 2016; Pokorny et al., 2017; Watts et al., 2017; Pollan, 2018)—functions that are often deficient in people with autism.

A further important difference between the psychedelic state and autism is that autism, like chronic schizophrenia, presents more as a trait than state-like phenomenon. In autism, the predictive coding architecture is skewed toward lower levels of the hierarchy, and critically, becomes set or fixed this way. It is hypothesized that lower levels of the functional hierarchy are ascribed greater precision in autism, which is often cast as a failure to attenuate sensory precision. This is thought to occur alongside a deficient weighting of high levels of the hierarchy, e.g., cognition linked to complex emotion and social awareness (Van de Cruys et al., 2014; van Schalkwyk et al., 2017). The psychedelic state, in contrast, is, by definition, not a trait phenomenon. Moreover, it is also richly emotional and empathic (Watts et al., 2017).

The psychedelic brain is likely to feature (at least in cases where the individual is healthy) a normally developed architecture that is transiently leveled, meaning that the brain’s functional hierarchy temporarily flattens. Once the psychedelic itself has been sufficiently metabolized, this temporary flattening reverts back to its original state. In brief, the hierarchical abnormalities in the psychedelic state are hypothesized to be transient, whereas those in autism appear to be relatively intransigent and biased to the lower (i.e., sensory) hierarchical levels.

#### 4. Other Disorders

Consistent explanations can be given for apparent phenomenological differences between other disorders such as Cotard delusion (Dieguez, 2018) and visual agnosia (Biran and Coslett, 2003), and the psychedelic state. Such disorders may, on first glance, appear to share some useful similarities with the psychedelic state, e.g., abnormal self-perception in Cotard delusion and an inability to group perceptual features in visual agnosia, yet they differ from the psychedelic state in the relative fixedness (Cotard delusion) and domain specificity of the relevant abnormalities.

#### 5. Meditative States

Meditative states are a special corpus of states that, traditionally, have been compared with the psychedelic state (Badiner and Gray, 2002). Psychological and neurobiological similarities and differences between different meditative states and the psychedelic state have recently been the focus of an extensive review paper (Milliere et al., 2018). One major psychological commonality is the relaxation of self-consciousness (Batchelor, 1998). The cessation of ego consciousness or attaining a state of “no self” is a core ideal of Buddhist philosophy and quality of effective meditation (Batchelor, 1998; Hanh, 2017). Importantly, the emergence of insight on cessation of ego-centered consciousness is another important component of Buddhist philosophy and practice, e.g., in Vipassana (i.e., Pali for insight meditation) (Batchelor, 1998). As we address in the forthcoming section, insight is also a core component of the psychedelic experience (Watts et al., 2017; Noorani et al., 2018).

In brief, key neurobiological similarities between meditative and psychedelic states that have been detected with brain imaging include the following: relative deactivation of the DMN (Carhart-Harris et al., 2012a; Garrison et al., 2013), reduced anticorrelation between the DMN and networks concerned with processing the extrinsic world (Carhart-Harris et al., 2013; Josipovic, 2014), and, recently, the enhancement of signatures of criticality (Atasoy et al., 2017). Important differences, such as the absence of decreased *α* power (Lomas et al., 2015) with meditation, may depend on the conventional use of relaxation techniques to enter a meditative state, although see Kakumanu et al. (2018) for evidence of increased brain entropy in experienced meditators practicing Vipassana meditation.

Psychedelics arguably induce ego dissolution much more reliably than most individuals’ meditations, albeit in a dose-dependent manner (Nour et al., 2016). As has been discussed above in relation to psychosis, improving our methods of capturing phenomenologically-rich states while recording brain activity may help to reveal consistencies between brain changes that have been observed with EEG and MEG and psychedelics and those known to occur in relevant nondrug-induced states. Such an approach does, however, present significant pragmatic challenges centered on the problem of the uncertainty principle, i.e., that in the act of measuring, we affect the phenomenon of interest, e.g., see Carhart-Harris (2018a) for a relevant discussion.

### F. Psychedelics and Insight

#### 1. Introduction

“This was a vision, fresh and clear as a mountain stream; the mind revealing itself to itself.”^18^

In this section, we address an assumption that is central to traditional psychedelic therapy, i.e., that psychedelics can facilitate psychological insight. Couched in the language of the dominant school of the day, psychoanalysis, clinicians conducting psychedelic therapy in the 1950s and 60s adhered to a model that maintained that these compounds work to relax the ego and its various defenses, such that unconscious material could emerge into consciousness. It is popularly commented that Freudian-minded therapists saw Freudian contents in their patients’ psychedelic experiences, whereas Jungians saw Jungian material, as if priming and biased perspective and interpretation rendered the relevant theories mere tautologies. This view may be too absolutist, however, as these deep psychological models may well share important similarities with each other and be much more complementary than contrasting. The same content may indeed be interpreted differently depending on the influence of different theoretical frameworks, but this does not preclude the possibility that the genuine release of unconscious, or, at least, incompletely conscious psychological material takes place via the antihierarchical action of psychedelics.

Previous work has sought to examine the notion that psychedelics facilitate the emergence of unconscious material into consciousness (in the classic psychoanalytic sense) using a primarily qualitative approach, supplemented by neurobiological data (Carhart-Harris, 2007). One must be wary of the fallacy of inverse inference, however, as neither isolated phenomenology nor neurobiology alone renders first-person experience real in the veridical sense. Thus, an improved methodology is required if this important principle of psychedelic therapy is to be better tested.

Fortunately, such developments are presently underway. Perhaps the most appealing is to measure insight by its behavioral consequences—an ironically Skinnerian approach—that is meritorious for its pragmatism. For example, acute experiences of self-reported insight were found to predict subsequent long-term clinical improvements (Carhart-Harris et al., 2018a) and changes in personality (Erritzoe et al., 2018) in individuals treated with psilocybin for treatment-resistant depression. Moreover, the same individuals became more accurate at predicting future life events after this treatment, meaning they were less delusionally pessimistic and thus more clear-sighted (Lyons and Carhart-Harris, 2018b). Separately, in a double-blind study with an active comparator (dextromethorphan), greater psychological insight was reported with psilocybin than the control compound (Carbonaro et al., 2018).

As with the replication of evidence, its convergence on a particular interpretation is another key indicator of its reliability and robustness—a process related to triangulation. Previous work has sought to demonstrate how numerous altered states, including dreaming (Carhart-Harris, 2007), exhibit a related phenomenology to the psychedelic state, including, of course, reports of the emergence of unconscious material into consciousness—alongside a related neurobiology (Carhart-Harris, 2007). However, we recognize that a stricter test of this principle will require the issuing of more structured assessments of insight and its consequences. The issuing of improved measures of insight has recently been addressed successfully in relation to dreaming (Edwards et al., 2013) and is now being implemented in our own psychedelic research.

#### 2. The Mechanics of Insight

What are the mechanics of insight? This matter has recently been approached from the perspective of the free-energy principle, both conceptually and computationally (Friston et al., 2017). Key concepts to invoke in this work include the following: 1) curious behavior, explorative search, novelty seeking, and epistemic learning, plus 2) structure and fact-free learning, abductive reasoning, Bayesian model selection (BMS),^19^ BMR,^20^ and “aha” or “eureka” moments. We will now unpack these various concepts and explain how they speak to the psychedelic experience.

It is widely recognized in literature on creativity that insight often occurs as part of a process, the initial phase of which involves an intention or plan, e.g., to discover something new. One heuristic for doing this is to relax one’s confidence in one’s prior assumptions (i.e., high-level priors), and, in so doing, promote an open, inquiring state of mind. This approach is essentially epistemic in nature, i.e., it is a behavioral strategy intended for learning, under the assumption that there is something to be learnt, i.e., there is some expected uncertainty (Friston et al., 2017).^21^ More than this, however, the knowledge seeker hopes to learn in an optimal way, e.g., learning as much as possible, with the least amount of effort. He/She does this by sampling areas where this is an opportunity to resolve a lot of uncertainty, i.e., where there is a lot to be learnt (Friston et al., 2017).

This is the essence of curious behavior (Friston et al., 2017) and is consistent with an explorative search strategy, which entails exploring novelty for the sake of significant knowledge gain (also known as intrinsic motivation in robotics) (Oudeyer and Kaplan, 2007; Schmidhuber, 2010; Barto et al., 2013). It is a strategy that dominates early in life and reduces later on, as exploitative searching becomes more relevant. Exploitative searching supersedes explorative search when the agent feels confident enough that some basic parameters have been established (Gopnik et al., 2017).

These heuristics for promoting insight resonate with those used by practitioners working therapeutically with psychedelics; e.g., a popular mantra told to patients ahead of recent trials with psilocybin is as follows: “trust, let go, be open” (Richards, 2015). Moreover, it has long been assumed, and also recently demonstrated, that approaching a psychedelic session with a clear therapeutic intention is conducive to subsequent positive mental health outcomes (Haijen et al., 2018). Perhaps relatedly, higher scores for trait absorption and lower scores for stubbornness have been found to predict consistent positive outcomes (Haijen et al., 2018). It is natural to intuit that these findings point to a common early-phase component of insight, in which the relaxation of prior assumptions (high-level priors), whether done willfully or with the assistance of a psychedelic, heightens an individual’s openness and receptivity to new perspectives.

The suggestion that the relaxation of (high-level) assumptions is conducive to insight is telling, as it implies that insight-related processes operate implicitly, i.e., without conscious awareness, and can be made more effective if executive function is suspended. It is worth noting that participants typically lie still with eyes closed during psychedelic treatment sessions and are advised to surrender their usual analytic thinking, in favor of a relaxed, freely-wandering mindset. This is consistent with itinerant processes characteristic of ordinary mind wandering—as well as dreaming—that are likely catalyzed further by psychedelics. Thus, rather than eagerly seeking out new data to resolve uncertainty, the agent is advised to forgo conventional epistemic foraging and instead allow processes to play out naturally and unconsciously, e.g., by sleeping on it (Friston et al., 2017) or doing something else.

The concept of structure learning (Tervo et al., 2016; Friston et al., 2017) is relevant to the phenomenon of insight. This refers to the identification of high-level structure or patterns in large, complex data sets, i.e., it emphasizes “seeing the woods” or the “bigger picture,” over getting “lost in the trees” or the details. An improved ability to see the “bigger picture” is logically implied by the energy landscape flattening effect of psychedelics discussed earlier. The notion of fact-free learning (Aragones et al., 2004; Friston et al., 2017) is also relevant. This refers to learning without necessarily accumulating new information or facts, i.e., because a fresh perspective or frame of reference, rather than more new data per se, may be more valuable for advancing understanding.

These concepts of structure and fact-free learning hint at something fundamental about the phenomenon of insight. Returning to the free-energy principle, we know that an agent can minimize free energy via perceptual (Friston and Kiebel, 2009) and active inference (Friston et al., 2012b), both of which entail sampling and sculpting the world to refine internal representations of it and thus reduce uncertainty or enhance confidence. There is, however, another higher level at which free energy may be minimized, namely via the selection and/or revision of beliefs about the acquired models themselves. This is known as BMS and BMR.

BMR is a particularly intriguing form of BMS that is likely to play a central role in brain development, e.g., in the form of synaptic pruning (Piochon et al., 2016) and formation of small-world architectures (Avena-Koenigsberger et al., 2014). In brief, BMR is the hypothesized mechanism via which high-level models are stripped of their redundancy so that simpler, more refined solutions may be revealed. Again, we see the theme of complexity minimization and compression in play. In this setting, one can refine high-level models or narratives to make them simpler by removing redundant parameters, thereby revealing the underlying core structures and manifolds. Crucially, this mechanism can proceed without the need for new data (fact-free learning) and is thought by some to be the purpose of sleep and accompanying synaptic homoeostasis (Hobson et al., 2014).

Importantly, optimal models must strike a balance between the following: 1) breadth or comprehension and 2) accuracy or precision. Broad (minimally complex) models can serve as catch-all representations that generalize across different scenarios. Meritorious for their generalizability, they may, however, suffer from oversimplicity, e.g., with too few parameters to say anything specific about a given phenomenon. Accurate models are naturally meritorious for their fidelity to a given phenomenon but can fall foul of overmodeling, in which an overparameterized model poorly translates from one phenomenon to another. It has been hypothesized that implicit processes of BMS and BMR underlie the experience of insight, with a particular emphasis on the latter. This is because the relevant “aha” or “eureka” experiences typically emerge spontaneously, “out of the blue,” as simple, elegant solutions, presumably because redundant models and/or model parameters have been unconsciously stripped away, leaving the “bare truth beneath.”^22^ Computational simulations have recently leant support to this conception of insight (Friston et al., 2017).

It is natural to speculate that BMS and BMR may underlie the occurrence of insight under psychedelics, and that psychedelics catalyze these processes of model refinement. Psychedelics have been shown to increase the entropy or complexity of spontaneous brain activity (Carhart-Harris, 2018a), which, on first glance, may be seen as inconsistent with BMS and BMR. Rather, we suggest that the acute psychedelic “hot state” reflects the preconditions for subsequent insight; i.e., it reflects a key phase of the process by which insight occurs in which confidence in high-level models is first relinquished so that content previously hidden from consciousness by the occluding influence of overly confident priors is now allowed to emerge, thereby enabling fresh perspectives to be entertained. Under conditions of relaxed high-level priors, disinhibited information is allowed to travel up the hierarchy and impress on consciousness as it does. In this way, one may be granted a fresh opportunity to cultivate changes to the relevant assumptions instantiated by high hierarchical levels.

Relatedly, in the acute hot state, the mind can explore its unfurled state space with fewer constraints, consistent with curious behavior (e.g., as in Fig. 1). In this way, the conscious explorer may discover novelty that was previously hidden from clear sight. Relatedly, he/she may report being able to see things from afar as per the overview effect originally described by astronauts (White, 1987). The overview effect is, of course, consistent with BMR and the pruning away of detail to see the bigger picture. Realizations of this sort are often felt as beautiful and transcendent in their simplicity and profundity but have also been queried as platitudinal:

“**Love is everything**… Is a platitude so deeply felt still just a platitude? No, I decided. A platitude is precisely what is left of a truth after it has been drained of all emotion. To resaturate that dried husk with feeling is to see it again for what it is: the loveliest and most deeply rooted of truths, hidden in plain sight. A spiritual insight? Maybe so” (Pollan, 2018).

The following are further firsthand references to experiences of insight experienced under psychedelics, plus another, for the sake of comparison, from an Apollo-mission astronaut. It is easy to appreciate their resonance with the mechanics described in this section, in particular the experience of being able to see a bigger picture:

“I was being reminded of things I already knew” (Watts et al., 2017); “I was learning without being taught” (Watts et al., 2017); “There had been, I felt, an opening of the heart” (Pollan, 2018); “Patrick described an epiphany having to do with simplicity: ‘…I was convinced in that moment I had figured it all out… It was right there in front of me… love… the only thing that mattered’” (Pollan, 2018); “Like google earth; I had zoomed out” (Watts et al., 2017); “In my cockpit window, every two minutes: the earth, the moon, the sun, and whole panorama of the heavens… And suddenly I realised that the molecules of my body, the spacecraft, the body of my partners, were all manufactured in some ancient generation of stars. I felt an overwhelming sense of oneness, or connectedness…– an insight, an epiphany” (Pollan, 2018).

#### 3. Criticality and Optimality

Criticality is a phenomenon described and studied within dynamical systems theory and complexity science that refers to complex dynamics that occur when a system nears a transition point between order and disorder. A self-organized critical system can exhibit complex emergent phenomena such as self-similarity, avalanching, long-range correlations, and critical slowing (Chialvo, 2010). The notion of a critical brain posits that neuronal activity is poised at criticality (Chialvo, 2004), and the entropic brain hypothesis states that psychedelics bring the brain closer still to such a critical point, as reflected by stronger signatures of criticality in the psychedelic state (Carhart-Harris et al., 2014; Carhart-Harris, 2018a). These latter findings imply that normal brain function (at least in awake adults) can be tuned closer to criticality, meaning that, with respect to the psychedelic state, normal waking consciousness biases order over disorder, or preservation over adaptation (Carhart-Harris et al., 2014; Carhart-Harris, 2018a)—at least in relation to the psychedelic state and even though it may still exhibit properties of criticality itself (Hilgetag and Hutt, 2014). The hypotheses that the waking brain can be tuned closer to criticality and that psychedelics do indeed do this have both recently been leant significant empirical support (Atasoy et al., 2017; Tagliazucchi, 2017; Muthukumaraswamy and Liley, 2018; Varley et al., 2019).

What is the relationship between criticality and model optimality? Are inequalities or asymmetries in the adult human brain’s functional architecture, such as high *α* power and high degree centrality of and metabolism in DMN nodes,^23^ indicative of a top-heavy system that biases high-level models (such as the ego and its various beliefs and defenses) over data? Does this top heaviness render the adult human mind somewhat impervious to the fullness of information contained within lower levels of the system, such as the classically mammalian system (MacLean, 1990) that is the limbic system? Is this top heaviness especially exaggerated in certain psychopathologies such as depression, obsessive-compulsive disorder, and eating disorders? These are examples of disorders that may rest on particularly rigid high-level priors that dominate cognition and likely serve a defensive function (Carhart-Harris, 2019).

These are important questions, and it is not difficult to imagine how they could be tested.^24^ Little work has been done on enduring brain changes after a psychedelic experience, but what evidence there is does suggest potential thinning in the posterior cingulate cortex node of the DMN with repeated ayahuasca use (Bouso et al., 2015) and also changes in DMN functional connectivity 1 day after psilocybin therapy for depression (Carhart-Harris et al., 2017). If BMR does play a role in insight under or after a psychedelic experience, we might predict a flattening of the relevant free-energy (attractor) landscape postacutely, e.g., this might present as an attenuated vestige of the more dramatic flattening likely to characterize the acute psychedelic state (Atasoy et al., 2018); e.g., see Fig. 1. Future work may focus on the DMN, with the prediction that its usual dominance is subdued post-treatment, e.g., with characteristic changes in time-series complexity and criticality that can be quantified empirically. If there is an enduring nature to the relaxation of high-level compressive models, we might also seek to test the notion that the brain is more sensitive to simple sensory prediction error post-treatment with a psychedelic. Such a finding would be consistent with a scenario in which a postacute relaxation of priors allows for an enhanced sensitivity to “data.” Recent findings of increased amygdala responsivness to emotional faces post-treatment with psilocybin, for example, could be seen as consistent with such a scenario (Roseman et al., 2018a). Other more classic prediction–error paradigms might also reveal an enhanced neuronal surprise signal post-treatment with psilocybin, consistent with a vestigial lightening of top-down constraints and concomitant sensitivity to bottom-up information flow.

#### 4. Therapeutic and Epistemic Transformation

There is growing interest in the potential of psychedelics to treat various psychiatric disorders (Carhart-Harris and Goodwin, 2017; Rucker et al., 2018). Evidence from a series of recent small-scale studies (Moreno et al., 2006; Grob et al., 2011; Gasser et al., 2014; Bogenschutz et al., 2015; Osório Fde et al., 2015; Carhart-Harris et al., 2016a, 2018a; Griffiths et al., 2016; Ross et al., 2016; Palhano-Fontes et al., 2019), in addition to population studies (Krebs and Johansen, 2013; Hendricks et al., 2015), controlled studies in healthy individuals (Griffiths et al., 2006; Schmid and Liechti, 2018), conceptual and empirical mechanistic work (Carhart-Harris et al., 2012a, 2014, 2017, 2018c; Carhart-Harris and Nutt, 2017; Roseman et al., 2018), naturalistic and observational studies (Thomas et al., 2013; Argento et al., 2017; Lafrance et al., 2017; Haijen et al., 2018), and meta-analyses of historic trials from the mid-20th century (Krebs and Johansen, 2012; Rucker et al., 2016) has leant collective support to the position that psychedelic therapy offers a promising new treatment option for patients and mental health care providers. The question now follows, how does it work (Carhart-Harris, 2019)?

Previous commentaries have hypothesized (Fletcher and Frith, 2009) and demonstrated aberrant predictive processing in a variety of psychopathologies, from autism (Van de Cruys et al., 2017) and schizophrenia (Powers et al., 2017) to addiction (Gu, 2018) and depression (Chekroud, 2015). Several factors support the notion that there exists a common mechanistic denominator underlying different expressions of mental illness. These include the following: 1) putatively high comorbidity (Maj, 2005), 2) the poor reliability of diagnoses across diagnosing clinicians (Richieri et al., 2011), and 3) absence of specific and reliable biomarkers to bolster such diagnoses (Venkatasubramanian and Keshavan, 2016), and the fact that the same category of drug can be used to treat a number of different disorders (Kramer, 1994; Whitaker, 2010). If such a common denominator exists, however, what might it be? Some have suggested it may be a loss of meaning and associated sense of dislocation (Alexander, 2008) or disconnection (Watts et al., 2017; Hari, 2018), others have proposed a narrowing of focus (or “capture”) in a context of a perceived loss of control (Kessler, 2016), while still others have proposed pathologic precision weighting of priors (Friston et al., 2014). See also Carhart-Harris (2019).

It is reasonable to suspect that these different models are largely inter-related, and, indeed, it is easy to see how they can be subsumed under the free-energy principle. In this study, we take the position that most, if not all, expressions of mental illness can be traced to aberrations in the normal mechanics of hierarchical predictive coding, particularly in the precision weighing of both high-level priors and prediction error. We also propose that, if delivered well (Carhart-Harris et al., 2018c), psychedelic therapy can be helpful for such a broad range of disorders precisely because psychedelics work pharmacologically (5-HT2AR agonism) and neurophysiologically (increased excitability of deep-layer pyramidal neurons) to relax the precision weighting of high-level priors (instantiated by high-level cortex) such that they become more sensitive to context (e.g., via sensitivity to bottom-up information flow intrinsic to the system) and amenable to revision (Carhart-Harris, 2018b).

Consistent with the model presented in this work, overweighted high-level priors can be all consuming, exerting excessive influence throughout the mind and brain’s (deep) hierarchy. The negative cognitive bias in depression is a good example of this (Beck, 1972), as are fixed delusions in psychosis (Sterzer et al., 2018).^25^ In this paper, we propose that psychedelics can be therapeutically effective, precisely because they target the high levels of the brain’s functional hierarchy, primarily affecting the precision weighting of high-level priors or beliefs. More specifically, we propose that psychedelics dose-dependently relax the precision weighting of high-level priors (instantiated by high-level cortex), and in so doing, open them up to an upsurge of previously suppressed bottom-up signaling (e.g., stemming from limbic circuitry). We further propose that this sensitization of high-level priors means that more information can impress on them, potentially inspiring shifts in perspective, felt as insight. One might ask whether relaxation followed by revision of high-level priors or beliefs via psychedelic therapy is easy to see with functional (and anatomic) brain imaging. We presume that it must be detectable, if the right questions are asked in the right way.

It seems reasonable to suppose that the relevant transformations reported under and after a psychedelic experience (Griffiths et al., 2006; Watts et al., 2017) are principally epistemic in nature, i.e., manifesting as revisions in awareness or perspective, but it stands to reason that they must still register on some level as a change in brain function. It may be difficult, but not impossible, to identify the relevant neuronal changes, and doing so will be a particular challenge for psychedelic science. One difficulty may relate to the level of analysis. For example, the apparently fundamental nature of the long-term psychological changes seen with psychedelics (e.g., in personality and outlook) suggests that something particularly high level or large scale may also have transpired in the brain.^26^ One thought is that we may need to look at trajectories of brain states rather than individual brain states per se, to see in high-level beliefs in action (Cabral et al., 2017), how they change after psychedelics (Lord et al., 2018), and how this relates to the relevant psychological change(s).

#### 5. Are Psychedelic-Induced Insights Trustworthy?

Before closing this section, we wish to address a difficult question that is often raised in relation to psychedelic therapy and specifically the principle that psychedelics work to facilitate insight. This is the question of whether the reported insight is veridical—in the sense of reflecting things that are actually true or real. For example, it is not uncommon for patients to report apparent recovery of repressed memories under psychedelics (Sandison, 1954; Carhart-Harris, 2007), and indeed, we had one such case in our recent psilocybin for treatment-resistant depression trial (Carhart-Harris et al., 2018a).

Without wishing to fall down a philosophical rabbit hole, the most pragmatic position may be to accept such experiences as psychologically real in the sense that they are inferences that serve to close an explanatory gap, and do so relatively effectively. In this sense, magical, religious, and delusional beliefs or interpretations may be considered psychologically real. That magical beliefs are common in situations of uncertainty (Bersabe and Arias, 2000) speaks to the principle that belief in magic can serve a psychological function. This sympathy with gap-filling, jumping to conclusions, or abductive reasoning (Friston et al., 2017) is also a principle that governs care in the context of patients with psychosis, for example, where clinicians refrain from attempting to correct delusional beliefs because of an acknowledgment that doing so may be destabilizing for the patient.

This problem has recently been addressed in a thoughtful philosophical piece, entitled “the epistemic innocence of psychedelic states” (Letheby, 2016), which essentially takes a stance that is consistent with the one described above, i.e., that (apparent) insight be viewed with equanimity if it appears to mediate positive therapeutic outcomes, even if the apparent insight is founded on dubious (e.g., supernatural) assumptions. Technically, the philosophical rabbit hole dissolves further in the face of the free-energy principle. This follows because the free-energy principle maintains that there is no absolute truth that is knowable absolutely—there is only evidence for a set of plausible hypotheses. In other words, the best beliefs or models are simply those with the greatest evidence—or minimum free energy.

In essence, the free-energy principle works to approximate reality through invoking, testing, revising, and optimizing models. Effective approximations of reality are reliable and trustworthy, whereas less optimal models are flimsy and fragile, e.g., they may be overly complex and/or poorly translatable. If such models fail, this will expose their fallibility to the agent, together with a likely unpleasant rise in his/her sense of uncertainty. Thus, a commitment to the discovery of better models is important, as improved models will provide more robust and reliable approximations to reality, which are likely to support greater stability in terms of mental health.^27^ These principles may be seen to speak to the merits of the scientific method for determining “what is best to believe.” For example, although supernatural belief systems may have once been helpful for humans^28^—they are arguably less so today. Moreover, beliefs held as a matter of faith are less amenable to the mechanics of self-correction that underlie empirical approaches.

### G. What to Do About the “woo”?^29^

Psychedelics have an interesting history of association with pseudoscience and supernatural belief. One interpretation of this is that a strong psychedelic experience can cause such an ontological shock that the experiencer feels compelled to reach for some kind of explanation, however tenuous or fantastical, to close an epistemic gap that the experience has opened up for them. This is an important matter, particularly as increasing numbers of people are likely to be experimenting with psychedelics in the coming years—but it is also a rich topic that deserves a separate discussion piece of its own.

It is important, however, to address this matter briefly in this work, as it is a problem that speaks to the special value of a naturalistic approach to psychedelics, as well as a responsible one that includes provision of education (e.g., about the importance of psychological preparation and integration) alongside careful engineering of the experience itself (Carhart-Harris et al., 2018c). If done properly, tenuous magical explanations can then be challenged appropriately (although not during the experience itself—as to do so would be inappropriate) in the skeptical, self-correcting fashion that is intrinsic to the scientific method. Reliable and robust models of natural phenomena, of the sort that science endeavors to discover and finesse, serve us best, as they are less likely to betray us, leaving us open to logical fallacies, dogmatism, absolutism, and an emotional and existential instability.

The phenomenon of spiritual bypassing is relevant in this work. This refers to an ontology in which individuals receive so much (potential) information, rapidly, without sufficient time to properly integrate or assimilate it (Masters, 2010). In experiencing such information overload, the psychedelic initiate may reach for bizarre beliefs or poorly understood platitudes, in an effort to explain away his/her felt uncertainty—in a similar way as may occur in the incipient phase of a psychotic disorder. Spiritual bypassing may be understood as an escapist defense, dressed up as a spiritual awakening. Combining psychedelic therapy with a secular wisdom teaching, such as can be found in nonreligious Buddhism for example (Batchelor, 1998; Hanh, 2017), as well as depth psychology (Freud, 1934, 1949; Jung, 1960, 1969), may have considerable value in this regard, helping to ground psychedelic science and medicine, while inoculating against evangelism.

### H. The Anarchic Brain

The anarchic brain principle is a natural counterpart to relaxed beliefs under psychedelics (REBUS). This is because the relaxation of high-level priors necessarily implies a liberation of bottom-up information flow (particularly from intrinsic sources). Thus, because top-down control is a defining property of hierarchy, REBUS is commensurate with a loss of functional hierarchy in the brain. The word “anarchy” has a Latin–Greek origin and means “without chief” or “ruler.” It is most typically used in political contexts to refer to “a state of disorder due to absence or nonrecognition of authority or other controlling systems” or “absence of government and [thus] absolute freedom of the individual” (Oxford English Dictionary). The anarchic brain principle is a close complement to the entropic brain hypothesis. Both feature as part of their definition, the principle that psychedelics enhance brain entropy (e.g., as measured by Lempel–Ziv complexity) and bottom-up information flow (e.g., measurable via effective connectivity). Both principles are also intended to apply first and foremost to spontaneous brain activity—and are difficult to assess using classic stimulus–response paradigms—for reasons discussed elsewhere (Carhart-Harris, 2018a).

As an extension of the anarchic brain principle, in this final section, we speculate that the structural organization of brains (and minds) recapitulates the organization of social systems. Just as the free-energy principle recognizes the importance of hierarchy inside the brain, so it recognizes it elsewhere in nature. For example, we may think of individuals constrained by communities, constrained by nation states, constrained by unions of states, etc. We may also reflect on the hierarchy of beliefs at each level, e.g., with individual beliefs constrained by community beliefs, constrained by national beliefs, constrained by transnational beliefs. We do not wish to take this analogy too far, only to raise the idea, which, of course, is inherent in the free-energy principle, that beliefs are hierarchical, and humans are apparently unique among the animal species because of the temporal thickness of their beliefs, spanning greater periods of time than do the generative models of other species (Becker, 2011; Chouraqui, 2011; Seth and Friston, 2016).

Grounding these ideas in biology, neuroimaging research with psychedelics has revealed a breakdown in modular integration under these compounds that is superseded by a desegregated, more globally interconnected organization of brain function (Roseman et al., 2014; Carhart-Harris et al., 2016c). It has also been shown that these brain changes correlate with peoples’ ratings of ego dissolution under the drug (Tagliazucchi et al., 2016), which, when it is surrendered to (Haijen et al., 2018), includes as a logical corollary of it, the so-called unitive experience, i.e., a sense of union and interconnectedness with objects and entities previously seen as external to oneself (Hanh, 2017; Roseman et al., 2018b). We have recently seen that a globally connected brain state is visited more regularly under psychedelics than other brain states, whereas certain high-level states/networks become neglected (Lord et al., 2018). Such an outcome can be seen as commensurate with a flattened energy landscape.

The unitive experience is closely related to the overview effect described in a previous section, and it is also the essential, defining characteristic of the mystical (Maclean et al., 2012)—or perhaps better-phrased peak experience (Roseman et al., 2018b). Note that whereas the peak experience itself can be treated as irreducible in the experiential sense, its occurrence may still depend on reaching a critical level of 5-HT2AR occupancy and signaling (Madsen et al., 2019)—a useful reminder that however high level a psychological phenomenon is, it will still rest on discernable physical, energetic, and chemical processes.

A number of research teams have shown that the occurrence of peak experiences under psychedelics is predictive of long-term positive changes in psychological well-being [e.g., Roseman et al. (2018b)], as well as changes in personality in the direction of increased openness (MacLean et al., 2011; Erritzoe et al., 2018). It is a matter of some curiosity that the personality trait openness has reliably been shown to correlate with liberal political perspectives (Carney et al., 2008), and a realization of this led to a series of studies by us (Nour et al., 2017; Lyons and Carhart-Harris, 2018a) and others (Forstmann and Sagioglou, 2017) that have demonstrated long-term changes in political perspective in association with the use of psychedelics.

It is natural to speculate from these findings that changes in brain function that may be defining of the psychedelic state, e.g., heightened brain entropy, modular disintegration, increased global integration, and increased bottom-up information flow from intrinsic sources (Carhart-Harris, 2018a), may be involved in the mediation of (potentially) fundamental transformations in beliefs, including changes in personality and outlook. Providing initial support for this, we previously found a relationship between increased brain entropy under LSD, as measured with functional magnetic resonance imaging, and subsequent increases in trait openness (Lebedev et al., 2016). Moreover, on a purely psychological level, we have found that ego dissolution mediates long-term increases in trait liberalism and decreased authoritarianism (Nour et al., 2017).

How these acute brain changes and associated mind states trigger the relevant long-term changes in beliefs is, at this stage, not fully understood—but increased bottom-up information flow (i.e., from lower-level intrinsic systems such as the limbic system) impacting on sensitized high-level priors (e.g., high-level networks and their dynamics) are logical places to look. The relevant transformations themselves are epistemic in nature, e.g., taking the form of a revised awareness or perspective. Once discovered, the newly appreciated awareness cannot easily be forgotten, tantamount in some ways to the effect of trauma. However, how do these epistemic transformations register in the brain? Are they detectable in anatomy as well as function? Is there such a thing as a liberal or libertarian brain (Kanai et al., 2011)? Is the brain’s functional hierarchy organized differently in an individual with strong libertarian and/or liberal views, compared with someone holding strong authoritarian and/or conservative beliefs?

Two figureheads in psychedelic research and therapy, Stanislav Grof and Roland Griffiths, have highlighted how psychedelics have historically “loosed the Dionysian element” (Pollan, 2018) to the discomfort of the ruling elite, i.e., not just in 1960s America but also centuries earlier when conquistadors suppressed the use of psychedelic plants by indigenous people of the same continent. Former Harvard psychology professor, turned psychedelic evangelist, Timothy Leary, cajoled that LSD could stand for “Let the State Dissolve” (Pollan, 2018). Whatever the interaction between psychedelic use and political perspective, we hope that psychedelic science will be given the best possible opportunity to positively impact on psychology, psychiatry, and society in the coming decades—so that it may achieve its promise of significantly advancing self-understanding and health care. An ill-thought–through repoliticization of psychedelic use would likely be counterproductive in this regard.

Whatever the sensitivities surrounding these issues, it seems right to openly acknowledge the possibility that increased psychedelic use could have broader social and political implications. If rates of psychedelic use were to escalate, poorly integrated experiences could leave individuals awash in uncertainty and eager for solace in tenuous, or, worse, delusional beliefs that serve to stop-gap uncertainty. We advocate a commitment to the position that—however otherworldly it may feel—the psychedelic experience is a thing of nature, intimately linked to discernible changes in brain function. It is possible that such a naturalistic, scientific approach to psychedelics may spawn greater interest in deep ecology (Naess, 1984). Relatedly, integrating observations from psychedelic science, with insights from physics and life sciences (Bejan, 2016), depth psychology (Safran, 2003; Hill, 2013), and Buddhist philosophy (Hanh, 2017), may help to nourish and ground the field.

### I. Summary

To summarize, the REBUS/anarchic brain model^30^ is derived from a synthesis of the entropic brain hypothesis and free-energy principle, and particularly the latter’s close links with hierarchical predictive coding. It recognizes that psychedelics induce an acute state of heightened brain entropy via their action on the serotonin system (Carhart-Harris, 2018a), then looks to prediction coding and hypothesizes that increased brain entropy reflects a relaxation of the precision weighting of priors (REBUS) that coincides with liberation of bottom-up signaling (anarchic brain).

To aid the testing of the REBUS/anarchic brain model, we propose a number of specific indices of priors and bottom-up signaling such as the functional integrity and dynamics of high-level brain networks (REBUS) and effective connectivity applied to relevant circuitry, such as circuitry connecting the hippocampus/parahippocampus with cortical nodes of the DMNs. More broadly, the model could be assessed by examining any of several aspects of hierarchy in the brain (Murray et al., 2014; Margulies et al., 2016; Deco and Kringelbach, 2017), with the simple hypothesis that key features of hierarchy will be diminished under a psychedelic.

We also dedicate significant discussion to implications of the REBUS/anarchic brain model. One of these is that the relaxation of priors implies that the system’s (context) sensitivity will be enhanced—as it will be more receptive to bottom-up information, particularly from intrinsic sources such as the limbic system. Thus, if the belief relaxation process is combined with good contextual support, pathologic beliefs underlying psychiatric illness, for example, may be amenable to revision during and after a psychedelic experience. The ideal effect of such a belief revision process would be to make the updated priors resonate more harmoniously with previously hidden or silenced information. If mediated properly, this belief–recalibration process may have long-term positive consequences for mental health; indeed, the described process is the very essence of effective psychedelic therapy (Haijen et al., 2018; Lyons and Carhart-Harris, 2018b).

Throughout this work, we have made reference to a prominent effect of psychedelics on hierarchically high-level priors. Although the REBUS model is not restricted solely to high-level priors, one compelling reason to place emphasis on hierarchically high or deep components of the brain is the especially high expression of 5-HT2ARs (the key target of psychedelics) on layer 5 pyramidal neurons within the brain’s high-level cortical regions (Jakab and Goldman-Rakic, 1998; Beliveau et al., 2017). Collectively, these high-level features of the brain are thought to encode expectations and beliefs about the lived world. Findings of dysregulated activity in pyramidal cell firing (Celada et al., 2008), the DMN (Carhart-Harris et al., 2012a), other high-level intrinsic networks (Lebedev et al., 2015; Carhart-Harris et al., 2016c), trajectories of brain states (Lord et al., 2018), and hierarchically high-level (but relatively low-frequency) spontaneous neuronal oscillations (Muthukumaraswamy et al., 2013) add further weight to the hypothesis that psychedelics have a particularly pronounced action high up in the brain’s functional hierarchy.

In accordance with the free-energy principle (and with dual-aspect monism), one can view the dynamics of high-level intrinsic networks as the dynamic or energetic counterparts of high-level priors (e.g., see Carhart-Harris and Friston, 2010). Indeed, the DMN itself has been described as the brain’s “Centre of Gravity” (Davey and Harrison, 2018). The self or ego is an important (perhaps central) high-level prior that DMN dynamics may instantiate (Carhart-Harris and Friston, 2010). The DMN is a particularly influential system, sitting atop a (uniquely) deep functional hierarchy in humans (Friston, 2018). We reason that high-level systems and the beliefs they encode have an informationally efficient, compressing influence on more elemental information held and processed below them. The high-level systems therefore function to route or canalize thought and behavior, sometimes in an overly constraining way that restricts self-awareness and obstructs new learning.^31^ If the gravitational pull of beliefs or behaviors becomes excessive, this can leave an agent feeling estranged from deeper aspects of his or her self, as well as from other people—and from a sense of purpose and meaning in life (Watts et al., 2017; Carhart-Harris et al., 2018b; Hari, 2018).

Curiously, psychedelics appear to temporarily breach the free-energy principle, i.e., by relaxing priors and increasing brain entropy for a time-limited period, but, once the drug has been sufficiently metabolized, normal brain function resumes and systems reset, potentially (but by no means absolutely) in a healthier way (ideally), more in tune with each other and the sensorium. We propose that mechanisms of BMS and BMR are catalyzed by the time-limited entropic state induced by psychedelics, such that model redundancy can be minimized, and high-level priors refined or “spring-cleaned.” The following is a first-person testimony that speaks to the hypothesized effect:

“It was like when you defrag the hard drive on your computer, I experienced blocks going into place, things being rearranged in my mind, I visualized as it was all put into order, a beautiful experience with these gold blocks going into black drawers that would illuminate and I thought: ‘My brain is bring defragged, how brilliant is that!’” (Watts et al., 2017).

Whether model reduction necessarily leaves an agent with a robust, reliable, and refined statistical representation of the world is, however, an open question—as is what the specific mechanics of this model reduction process are, and how we can measure them in the brain.

Returning to psychology, in the previous section we addressed a phenomenon known as spiritual bypassing that can be observed among some (Western)^32^ users of psychedelics. Magical beliefs may be helpful (via reducing free energy and associated uncertainty), particularly in cultures that support this, but it is difficult to see how the use of any belief as psychological escape can be regarded as optimal or sustainable. We anticipate that it may become possible in the future to demonstrate, via some particular metric, how harmony between different levels of the brain’s functional hierarchy relates to a felt psychological harmony reflective of a well-integrated mind and brain. Doing so may require that we look at longitudinal data such as mood stability (and associated brain activity) over months, with the prediction that emotional escape or avoidance predicts long-term mood instability, even after a potential initial positive spike in mood. Although speculative, candidates for meaningful metrics of harmony in the brain/body may be found in the various signatures of criticality, such as long-range correlations, nesting of oscillatory rhythms (Murray et al., 2014), body–brain physiologic coupling (Richter et al., 2017), and environment–brain alignment (Lakatos et al., 2013). This notion of harmony may also relate to a previously discussed construct of connectedness and its special relevance to psychedelic therapy (Watts et al., 2017; Carhart-Harris et al., 2018b).

## III. Conclusions

This paper has sought to marry insights from the free-energy principle with those of the entropic brain hypothesis to account for the acute and longer-term brain and mind effects of psychedelics. We have named this synthesis REBUS and the anarchic brain. “REBUS” is an intentionally easy-to-remember acronym based on the key theme of RElaxed Beliefs Under pSychedelics, and the anarchic brain follows naturally from the liberation of bottom-up signaling—which is logically implied by relaxed precision on priors. We have described how this mechanism begins with agonist actions at the 5-HT2AR and can be reflected in the entropy of spontaneous cortical activity. The present model significantly advances on the entropic brain hypothesis, however, by introducing the notion of a relaxation of the precision weighting of priors—which concomitantly liberates bottom-up signaling that, with the right preparedness, care, and aftercare, can aid the revision of pathologically overweighted priors.

We propose that the REBUS/anarchic brain model can explain the full gamut of phenomena associated with the psychedelic experience, including the emergence of previously unconscious psychological material into conscious awareness.^33^ We also propose that high-level priors confer a broad summarization of the mind and world, effectively suppressing away (potential) content. It therefore follows that if this suppression is relaxed, as it is under psychedelics, content will necessarily be released. It is an ideal of psychedelic therapy that this newly available content be appropriately integrated and assimilated into existing mental models so that more of the inner and outer world can be processed.

Importantly, we also propose that the acute brain conditions induced by the pharmacological action of psychedelics, increasing brain entropy and relaxing the precision weighting on priors, are ideal for working on the revision of high-level priors or beliefs. Consistent with recent work on the mechanics of insight, an entropic brain with relaxed high-level priors will be more amenable to curious behavior and Bayesian model optimization processes that can aid the occurrence of insight and perspective change. We suspect that this template of pharmacologically and contextually mediated belief relaxation and revision may serve a major role in the future of mental health care, potentially improving the efficacy of current treatment strategies by factoring in contextual variables when assessing the neurobiology of a given disorder and the viability of a specific intervention (Carhart-Harris, 2018b).

Finally, although we are optimistic about the potential of psychedelic therapy to positively impact on psychology and psychiatry in a major way in the coming decades, it feels prudent to consider complications that may arise as increasing numbers of people choose to undergo a psychedelic experience. In this study, we wish to highlight what the scientific model has to offer, i.e., via trial and error, forever improving models of mind and world. If integrated properly, psychedelics have every chance of becoming a legitimate, if not lauded, tool of science and medicine—capable of awakening us to the true depths of our being and continuousness with nature.

“Whether by their very nature or the way that first generation of researchers happened to construct the experience, psychedelics introduced something deeply subversive to the West that the various establishments had little choice but to repulse. LSD truly was an acid, dissolving almost everything with which it came into contact, beginning with the hierarchies of the mind… and going on from there to society’s various structures of authority and then to lines of every imaginable kind… If all such lines are manifestations of the Apollonian strain in Western civilisation, the impulse that erects distinctions, dualities, and hierarchies, and defends them, then psychedelics represented the ungovernable Dionysian force that blithely washes all those lines away… But surely [it] is not the case that the forces unleashed by these chemicals are necessarily ungovernable” (Pollan, 2018).

## Footnotes

1 REBUS is an acronym for “RElaxed Beliefs Under pSychedelics.”

2 The phenomenon of free energy, as it relates to brain function, was first introduced by Karl Friston in 2005 [Friston K (2005) A theory of cortical responses. *Philos Trans R Soc Lond B Biol Sci* 360(1456):815–36] and later developed into the “free-energy principle” [Friston K, Kilner J, Harrison L (2007) A free-energy principle for the brain. *J Physiol Paris* 100(1–3):70–87]. The free-energy principle is closely related to hierarchical predictive coding and the Bayesian Brain. For comprehensive texts on these models, see: (Andy Clark, *Surfing Uncertainty*) and [Hohwy J (2014) *The Predictive Mind*]—and here: [Tappin BM, Gadsby S (2019) Biased belief in the Bayesian brain: A deeper look at the evidence. *Conscious Cogn* 68:107–114] for a useful critique and indication of the robustness of the model in the context of psychotic delusions. As a simple nontechnical rule, free energy can be thought of as a measure of surprise. Thus, it is intimately related to information theoretic entropy, which is a measure of uncertainty or average surprise.

3 The entropic brain hypothesis (EBH) was first introduced in 2014 (Carhart-Harris et al., 2014) and later revisited and updated in 2018 (Carhart-Harris, 2018a). A stern test of the EBH would be to assess the specificity of the relationship between brain entropy and richness of conscious content, and one way to do this would be to assess the question of whether stimulant drugs, which increase arousal but presumably not conscious content, increase brain entropy. If they do increase brain entropy (to a similar extent to psychedelics), then this would contradict and potentially falsify the EBH.

4 Precision is formally inverse variance and can be thought of as synonymous with felt confidence.

5 Technically, a loss of precision (confidence) in posterior beliefs corresponds to a decrease in the curvature or flattening of the free-energy landscape associated with the neuronal activity encoding those beliefs. Free-energy minimization effectively tries to find flat minima in accord with Occam’s principle and Jayne’s principle of maximum entropy (Jaynes, 1957). Interestingly, the problem of sharp minima is still an area of active research in machine learning (Hochreiter and Schmidhuber, 1997) Flat minima lend neuronal activity a latitude for fluctuations that characteristically result in critical slowing, of the sort entailed by the entropic brain hypothesis. The “slow” in critical slowing can be thought of as a slow recovery from a perturbation, i.e., the system has a slower recovery rate. See Friston et al. (2012a) for a technical discussion.

6 Physiologically, precision is thought to be encoded by the sensitivity or postsynaptic gain of neuronal populations encoding predictions and prediction errors. Generally, a loss of precision corresponds to an increased sensitivity to afferent, presynaptic input. In turn, this enables revision or updating of beliefs that are neuronally encoded. See Kanai et al. (2015).

7 Note: we do not differentiate between different types of ego dissolution. For example, it has been argued that ego dissolution can be as follows: 1) incomplete: due to psychological resistance and/or insufficient drug dose, ensuing ego dissolution is experienced as a state of high anxiety and distress or 2) complete: felt as a state of complete surrender, associated bliss, and union with all things. We are currently developing a relevant emotional breakthrough inventory rating scale to better differentiate between these two types of ego dissolution. An argument could be made that only the latter is true ego dissolution, as it is an almost binary, qualitative phenomenon, i.e., it either happens or it does not. It is obviously of interest to better understand the neuronal correlates of these distinct experiences. Perhaps relevantly, recent positron–emission tomography imaging of 5-HT2AR occupancy and its relationship to subjective effects supports the assumption that the dose–response curve for psychedelics is nonlinear: Madsen et al. (2019).

8 Not discounting the relevance of differential sensitivities or vulnerabilities to the effects of injury, such as the role of genetic risk factors.

9 And perhaps individuals living within technologically and industrially advanced societies.

10 Technically, this imperative is identical to that used in predictive coding and variational free-energy minimization, namely, the compression of information via the minimization of redundancy or complexity (Hinton and Zemel, 1993; MacKay, 1995; Schmidhuber, 2010).

11 For a more detailed account of the specific signalling pathways engaged by psychedelics, see Marek (2018) and [López-Giménez JF, González-Maeso J (2018) Hallucinogens and serotonin 5-HT(2A) receptor-mediated signaling pathways. *Curr Top Behav Neurosci* 36:45–73]. Do note that these signalling pathways are complex as it is still not yet fully clear which signalling pathway or combination of them is necessary and sufficient for a psychedelic drug’s typical effects to manifest. There is also the phenomenon of functional selectivity to consider, i.e., that psychedelics appear to activate 5-HT2ARs in a different way to the endogenous ligand, 5-HT [González-Maeso J, Sealfon SC. Agonist-trafficking and hallucinogens. *Curr Med Chem* 16(8):1017–27]. The context and location of the 5-HT2AR–expressing neuron also needs to be factored in, e.g., highly 5-HT2AR–expressing deep-layer pyramidal neurons appear to be those that are most likely to be directly activated by psychedelics [Martin DA, Nichols CD (2016) Psychedelics recruit multiple cellular types and produce complex transcriptional responses within the brain. *EBioMedicine* 11:262–277].

12 We use the metric of within-network functional connectivity as an index of integrity within the DMN here.

13 The “critical” here refers to a “critical point” in a phase transition between order and disorder in which interesting emergent phenomena such as critical slowing become evident; Chialvo (2010).

14 Critical slowing refers to a property of critical systems in which they are especially sensitive to perturbation. The term slowing refers to the property of being slow to recover from the perturbation—analogous to a pebble causing persistent ripples on the surface of a lake—versus, e.g., a pebble having little impact on a thickly frozen (subcritical) lake.

15 Ketamine has similarly been shown to have a rapid mood-lifting effect in depression; however, its ability to engender lasting psychological changes does not appear to be equivalent to that of the classic psychedelics. More work is needed to ascertain why this is the case, but one possibility is that (perhaps due to an absence of a significant gradient in the cortical–subcortical *N*-methyl-D-aspartate receptor profile) ketamine does not subvert hierarchical message passing in quite the same way as psychedelics, and thus the release of (precise) bottom-up influences on sensitized high-level priors—leading to their long-term revision—is less evident with ketamine. Relatedly, ketamine may not engage insight-related processes in the same way as classic psychedelics appear to.

16 Indeed, there is comparatively little research on psychedelics affecting truly higher-level priors, although we looking to rectify this.

17 Note an important nuance here: hypersensitivity to stimulation extrinsic to the system (i.e., via sensory stimulation) may only be the rule within a critical dose range, above which brain function is likely to be dominated by its intrinsic activity—and so become insensitive to extrinsic information (Michaiel et al., 2019), but still sensitive to bottom-up information flow from within the system itself, e.g., via the limbic system to the cortex.

18 https://www.youtube.com/watch?v=kjeCNnbVYAQ.

19 BMS is a mechanism by which a best-performing model is identified from a set of models. Model performance is determined by a trade-off between model fit (specificity) and model complexity. Good models are those that fit data well enough, i.e., they do not overfit the data through the inclusion of too many parameters.

20 Bayesian model reduction is a mechanism through which model parameters are pruned to reduce model complexity. It is a mechanism of model optimization done in aid of model selection.

21 Expected uncertainty is a mindset that is encouraged ahead of psychedelic therapy and speaks to an important difference between the psychedelic experience and psychosis, i.e., where uncertainty is not expected and so prediction error is not accepted or integrated.

22 Addressing the question of whether the unconscious in the classical psychoanalytic sense (regardless of specific schools) influences conscious cognition and behavior, we can answer that it is our assumption that the unconscious does indeed influence behavior. This assumption is consistent with the position of classical psychoanalytic thinkers such as Freud and Jung. Personal testimonies speak to this assumption when individuals report insights about how past events have led to current behaviors without their conscious awareness. It is clearly important to consider how this assumption might be tested, so that stronger inferences can be drawn. One might construe implicit processing as a cognitive psychology homologue of this psychoanalytic idea, and implicit association paradigms could be used to probe it behaviorally.

23 Marcus Raichle: “Chaos is averted because all systems are not created equal… At the top of hierarchy resides the DMN” —cited in Ref. 20.

24 First one needs to provide a concrete definition of top heaviness and then test, e.g., that this is greater in relevant psychopathologies versus health and also that effective treatment with a psychedelic reduces them. One candidate for top-down influence might be DMN to limbic effective connectivity; another is *α* power; and yet another is to equate top heaviness with subcriticality, and thus state that stronger signatures of criticality reflect a less top-heavy system.

25 To be clear, for reasons stated earlier, we are not, at this stage, proposing psychedelic therapy as a treatment of psychotic disorders. Such a proposition would require much careful thought and qualification—and even still, may not pass as feasible/indicated.

26 Note also that preclinical molecular and cellular approaches can now detect long-term postpsychedelic changes, as might human positron–emission tomography and high-field magnetic resonance imaging. Although it is not always straightforward to decipher how molecular and cellular changes translate to human psychology, combining high-resolution measures with conventional functional brain imaging may be a potentially fruitful strategy.

27 From a statistical perspective—and machine learning—this robustness is cast in terms of the ability to generalize to new data. This rests on flattening short minima to reduce model complexity and preclude overfitting.

28 Note: we adopt the view that Buddhism (like science) is not a religion but a method and (like science) performs best when treated as such.

29 “woo” or “woo-woo” is a slang term with an unknown etymology. It is assumed to stem from its jocular and often pejorative use, when imitating a sound effect or the music one might hear during the depiction of supernatural events on television, in theatre, or in films. Succinctly, “woo-woo” refers to belief in the supernatural, paranormal, occult, and has a particular association with pseudoscience, particularly in the West.

30 We treat the two components as mutually dependent, i.e., relaxed priors naturally imply increased prediction error.

31 One might think of the notion of the “ego tunnel” here (Metzinger, 2009).

32 One could argue that spiritual bypassing is a distinctly Western phenomenon, as developments in scientific understanding in the West means that (unlike among indigenous tribes for example) the dominant culture is no longer set up to support magic. One may choose to ignore or reject scientific knowledge in the West, but to do so is contrary to evidence. Conversely, among indigenous cultures, magical explanations may be the best explanations available, and thus, no such accusations of willful ignorance can be made against these cultures.

33 Consistent with basic tenets of psychoanalytic theory and Buddhist teaching, we adopt the position that much of the mind is indeed unconscious and what is kept from conscious awareness can be personal and collective as well as “once conscious—but now repressed” or simply “never conscious.” We also maintain the unconscious often (but not always) drives conscious behavior in the absence of conscious awareness, in the way that a horse can steer its rider, without the rider realizing.

## Abbreviations

5-HT2AR: serotonin 2A receptor
BMR: Bayesian model reduction
BMS: Bayesian model selection
DMN: default-mode network
EEG: electroencephalogram
HPPD: hallucinogen-persisting perceptual disorder
LSD: d-lysergic acid diethylamide
MDMA: *3,4-methylenedioxy-methamphetamine*
MEG: magnetoencephalography
REBUS: relaxed beliefs under psychedelics
